# Biomimetic nanoparticles deliver mRNAs encoding costimulatory receptors and enhance T cell mediated cancer immunotherapy

**DOI:** 10.1038/s41467-021-27434-x

**Published:** 2021-12-14

**Authors:** Wenqing Li, Xinfu Zhang, Chengxiang Zhang, Jingyue Yan, Xucheng Hou, Shi Du, Chunxi Zeng, Weiyu Zhao, Binbin Deng, David W. McComb, Yuebao Zhang, Diana D. Kang, Junan Li, William E. Carson, Yizhou Dong

**Affiliations:** 1 Division of Pharmaceutics & Pharmacology, College of Pharmacy, The Ohio State University, Columbus, OH 43210 USA; 2 State Key Laboratory of Fine Chemicals, Dalian University of Technology, Dalian, China; 3 Center for Electron Microscopy and Analysis, The Ohio State University, Columbus, OH 43212 USA; 4 Department of Materials Science and Engineering, The Ohio State University, Columbus, OH 43210 USA; 5 Department of Surgery, Division of Surgical Oncology, The Ohio State University Wexner Medical Center and The OSU James Comprehensive Cancer Center, Columbus, OH USA; 6 Department of Biomedical Engineering, The Ohio State University, Columbus, OH 43210 USA; 7 The Center for Clinical and Translational Science, The Ohio State University, Columbus, OH 43210 USA; 8 The Comprehensive Cancer Center, The Ohio State University, Columbus, OH 43210 USA; 9 Dorothy M. Davis Heart & Lung Research Institute, The Ohio State University, Columbus, OH 43210 USA; 10 Department of Radiation Oncology, The Ohio State University, Columbus, OH 43210 USA

**Keywords:** Drug delivery, Immunization, Tumour immunology

## Abstract

Antibodies targeting costimulatory receptors of T cells have been developed for the activation of T cell immunity in cancer immunotherapy. However, costimulatory molecule expression is often lacking in tumor-infiltrating immune cells, which can impede antibody-mediated immunotherapy. Here, we hypothesize that delivery of costimulatory receptor mRNA to tumor-infiltrating T cells will enhance the antitumor effects of antibodies. We first design a library of biomimetic nanoparticles and find that phospholipid nanoparticles (PL1) effectively deliver costimulatory receptor mRNA (CD137 or OX40) to T cells. Then, we demonstrate that the combination of PL1-OX40 mRNA and anti-OX40 antibody exhibits significantly improved antitumor activity compared to anti-OX40 antibody alone in multiple tumor models. This treatment regimen results in a 60% complete response rate in the A20 tumor model, with these mice being resistant to rechallenge by A20 tumor cells. Additionally, the combination of PL1-OX40 mRNA and anti-OX40 antibody significantly boosts the antitumor immune response to anti-PD-1 + anti-CTLA-4 antibodies in the B16F10 tumor model. This study supports the concept of delivering mRNA encoding costimulatory receptors in combination with the corresponding agonistic antibody as a strategy to enhance cancer immunotherapy.

## Abstract (web summary)

Antibodies targeting OX40 or CD137, two T cell costimulatory receptors, have been shown to improve antitumor immunity. Here the authors design a phospholipid-derived nanoparticle to deliver OX40 or CD137 mRNA to T cells in vivo, improving efficacy of anti-OX40 and anti-CD137 antibody therapy in preclinical tumor models.

## Introduction

**Fig. 1:**
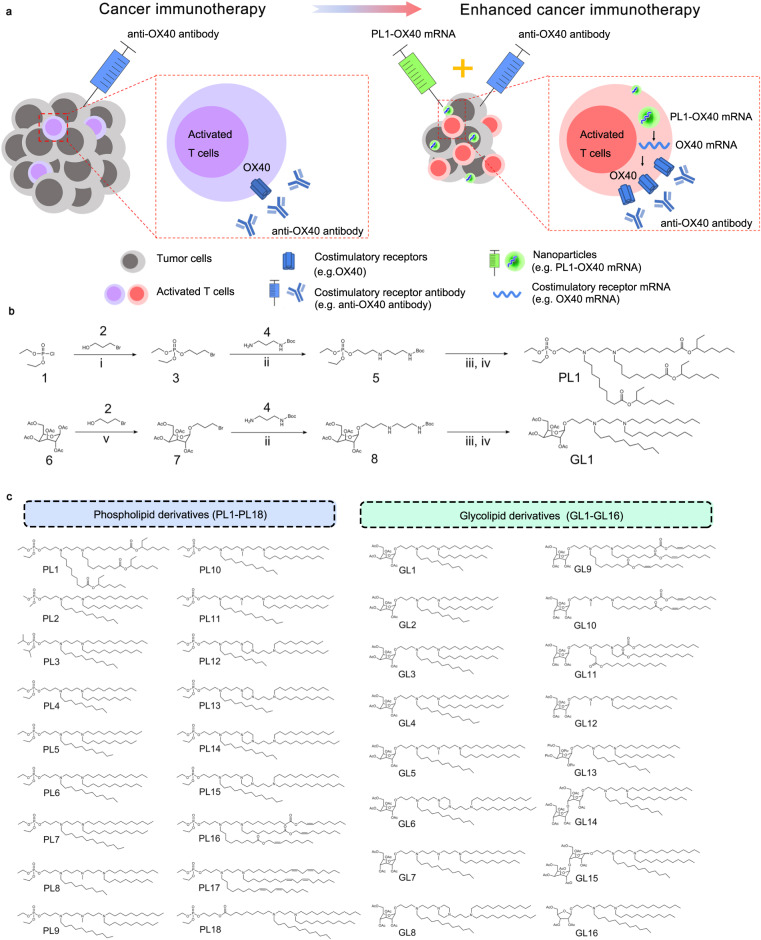
Stimulation of T-cell-mediated cancer immunotherapy. **a** Illustration of enhanced cancer immunotherapy via nanoparticles delivering costimulatory receptor mRNA followed by the injection of agonistic antibodies to costimulatory receptors (e.g., PL1-OX40 mRNA + anti-OX40 antibody). **b** Representative synthetic routes to biomimetic compounds: phospholipid and glycolipid derivatives. i. Et_3_N, Toluene, RT. ii. Et_3_N, DMF, RT. iii. TFA, CH_2_Cl_2_, RT. iv. aldehyde, Et_3_N, THF, NaBH(OAc)_3_. **c** Structures of phospholipid derivatives PL1-PL18 and glycolipid derivatives GL1-GL16.

Cancer immunotherapy encompasses a variety of approaches to stimulate antitumor immune responses^1–5^, including cancer vaccines^6–8^, cell-based therapies^9–12^, immune checkpoint blockade^13–15^, monoclonal antibodies^16,17^, mRNA based immunotherapies^18–20^, and nanoparticle-mediated immunotherapies^21–24^. In particular, the use of immune checkpoint inhibitors has led to improved overall survival for many cancer patients by targeting the T-cell co-inhibitory pathways such as PD-1 and CTLA-4^25^. Although these antibodies are used routinely in the clinic, the percentage of patients that experience a meaningful tumor response is only about 25%^26^. Therefore, there is an urgent need to develop safe and effective immunotherapy strategies for cancer treatment. Previously, researchers discovered a series of costimulatory molecules on T cells for cancer immunotherapy^27–29^. The cell-surface interactions between the ligands of costimulatory molecules and their costimulatory receptors activate clonal T-cell expansion and differentiation, thus leading to increased antitumor efficiency in several human cancers^27^. CD137 (also known as 4-1BB) and OX40 (also known as CD134) are T-cell costimulatory receptors and provide activating signals for CD8 and CD4 T cells^30,31^. CD137 plays an important role in T-cell proliferation and cytokine secretion. Recently, two agonistic anti-CD137 antibodies (urelumab and utomilumab) have been investigated in clinical trials^14^. OX40 is involved in stimulating CD8+ T cells for the generation of antitumor immune responses^32,33^. Agonistic anti-OX40 antibodies augment T-cell differentiation, cytolytic function, and antitumor immunity in various cancer types. Several agonistic anti-OX40 antibodies are currently studied in clinical trials^32,34,35^. Although costimulatory signals are critical to T-cell stimulation, these molecules are not always expressed at high levels within the tumor microenvironment, which impedes immunotherapeutic treatments^27^. Therefore, we hypothesized that delivering costimulatory receptor mRNAs increases their expression on tumor-infiltrating T cells, and when coupled with the use of corresponding agonistic antibodies, this treatment regimen directly activates T cells and improve cancer immunotherapy (Fig. 1a).

Here, we demonstrate that one phospholipid-derived nanoparticle, PL1, efficiently delivers mRNA to T cells both in vitro and in vivo. The combination of PL1-OX40 mRNA and anti-OX40 antibody exhibits significantly improved antitumor activity compared to anti-OX40 antibody alone in multiple tumor models. Moreover, this treatment approach significantly improves the immunotherapeutic effect of anti-PD-1 + anti-CTLA-4 antibodies. Mechanistic studies indicate that the delivery of PL1-OX40 mRNA could induce the activation of various immune cells, including T cells and dendritic cells (DCs). This study provides a platform to deliver costimulatory receptor mRNA to immune cells in order to boost antitumor immunity.

## Results

### Design and synthesis of phospholipid and glycolipid derivatives (PLs and GLs) for mRNA delivery

To test the hypothesis (Fig. 1a), we first need to deliver costimulatory receptor mRNA into T cells. We leveraged the unique characteristics of the phospholipids and glycolipids that are natural components of the cell membrane^36^. Based on their chemical structures, we designed and synthesized a library of phospholipid and glycolipid biomimetic materials, which are composed of a biomimetic head (phosphate head or glyco head), an ionizable amino core, and multiple hydrophobic tails (Supplementary Fig. 1). These phospholipid and glycolipid derivatives (PLs and GLs) were synthesized according to previously reported procedures^37,38^. Figure 1b shows representative synthetic routes to PL1 and GL1. Following this synthetic route, we synthesized PL1-18 and GL1-16 materials (Fig. 1c), which were characterized by ^1^H nuclear magnetic resonance (NMR) and mass spectroscopy (MS) (Supplementary Methods). Next, PLs and GLs nanoparticles were formulated with firefly luciferase mRNA (FLuc mRNA)^39–41^, and their size, surface charge, and mRNA encapsulation efficiency were characterized (Supplementary Fig. 2a–c). Since these compounds were ionizable lipids containing 2–4 tertiary amines, they were positively charged in the formulation buffer (pH = 3). We speculated that both the number of tertiary amines and the chain structure in the lipid derivatives would affect their interactions with mRNA.

**Fig. 2:**
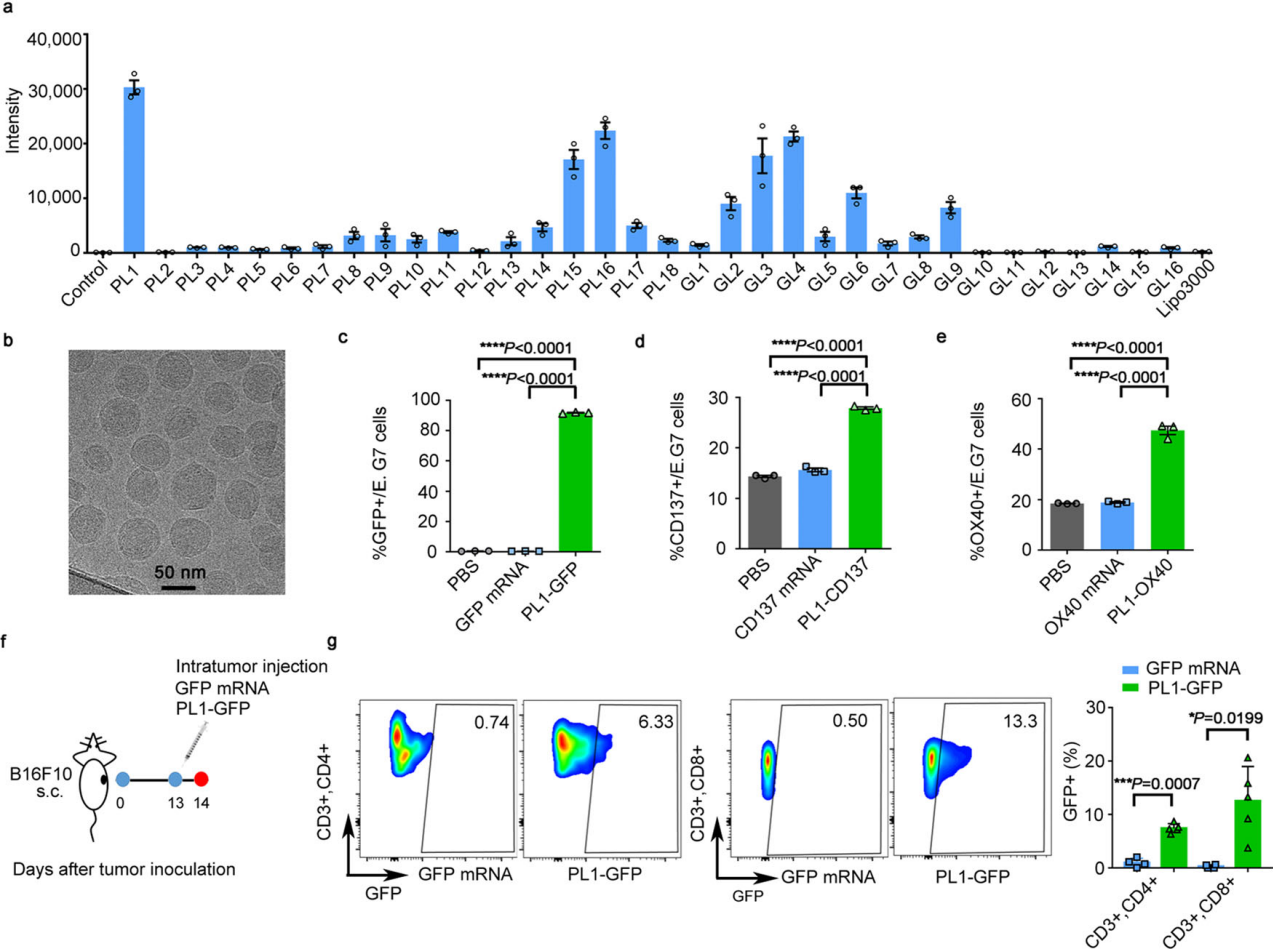
Biomimetic phospholipid- and glycolipid-derived nanoparticles for mRNA delivery. **a** Luminescence intensity of phospholipid- (PL) and glycolipid-derived (GL) nanoparticles delivering firefly luciferase (Fluc) mRNA to E.G7 cells. **b** Cryo-TEM image of PL1-mRNA nanoparticles (a representative image from one of five independent fields of view in a single experiment). Scale bar = 50 nm. **c** PL1 nanoparticles delivered GFP mRNA to E.G7 cells. **d** PL1-CD137 induced CD137 expression in E.G7 cells. **e** PL1-OX40 induced OX40 expression in EG.7 cells. **f** Scheme of GFP expression in B16F10 tumors after a single injection of free GFP mRNA or PL1-GFP. **g** GFP expression in CD4+, CD8+ T cells, after a single intratumoral injection with GFP mRNA (*n* = 4) or PL1-GFP mRNA (*n* = 5) in B16F10 tumors. Data in **c**, **d** and **e** are representative of three independent experiments. All data are presented as the mean ± S.E.M. Statistical significance in **c**, **d**, **e** was analyzed using one-way ANOVA followed by Dunnett’s multiple comparison test, and data in **g** was analyzed using the two-tailed Student’s *t*-test. *P < 0.05; ***P < 0.001; ****P < 0.0001. Source data are provided as a Source Data file.

Then, we studied the mRNA delivery efficiency of PL1-18 and GL1-16 nanoparticles in E.G7 cells (a T-lymphocyte cell line) and found PL1 nanoparticles displayed the highest delivery efficiency of FLuc mRNA (Fig. 2a). PL1-mRNA nanoparticles are homogenous in size, as visualized from the cryo-TEM image (Fig. 2b). Both RiboGreen and gel electrophoresis assays showed high encapsulation of mRNA in PL1 nanoparticles (Supplementary Figs. 2c and 3). Moreover, PL1 delivered GFP mRNA to about 90% of E.G7 cells, demonstrating its potential as a T-cell delivery vehicle (Fig. 2c, and Supplementary Fig. 4a). Then, we delivered the T-cell costimulatory receptor CD137 and OX40 mRNA to E.G7 cells. Flow cytometry results showed that both PL1-CD137 mRNA and PL1-OX40 mRNA significantly increased the cell-surface expression of CD137 (27.8%) and OX40 (47.4%), respectively (Fig. 2d, e and Supplementary Fig. 4b, c). We further investigated endocytic pathways of the PL1 nanoparticles using endocytic inhibitors, including 5-(*N*-Methyl-N-isopropyl)amiloride (EIPA) for macropinocytosis, chlorpromazine hydrochlorides (CPZ) for clathrin-mediated endocytosis, and methyl-beta-cyclodextrin (MβCD) for caveolae-mediated endocytosis. Treatment with EIPA, CPZ, and MβCD significantly inhibited the cellular uptake of PL1 nanoparticles by 50%, 56%, and 39%, respectively (Supplementary Fig. 5). This indicates that PL1 nanoparticles were internalized through multiple endocytic pathways.

Since PL1 nanoparticles accumulate mostly in the tumor after intratumoral injection (i.t.) in a subcutaneous B16F10 tumor model (Supplementary Fig. 6), we investigated the uptake of PL1 nanoparticles in tumor-infiltrating lymphocytes via an i.t. injection of PL1-GFP (Fig. 2f). We observed increased GFP expression in tumor-infiltrating CD4+ and CD8+ T cells (Fig. 2g), as well as in macrophages and dendritic cells (DCs) (Supplementary Fig. 7a–c). Consistently, increased OX40 expression was also observed in tumor-infiltrating lymphocytes after i.t. injection of PL1-OX40 (Supplementary Fig. 7d–g). Based on these results, PL1 nanoparticles were chosen for delivering CD137 and OX40 mRNA in vivo.

### Regression of tumor growth with the treatment of PL1-CD137 mRNA + anti-CD137 antibody

We then performed intratumoral injection of PL1-CD137 in combination with anti-CD137 antibody every other day for six doses in the B16F10 melanoma mouse model. Administration of PL1-CD137 + anti-CD137 Ab dramatically decreased the tumor growth rate by 5-fold (18 days after inoculation) (Supplementary Fig. 8a, b), and increased the overall survival time compared to PBS and PL1 (empty nanoparticle) + anti-CD137 Ab (Supplementary Fig. 8c). Similar experiments were conducted in the A20 lymphoma tumor model. Treatment with PL1-CD137 + anti-CD137 Ab resulted in a 2-fold decrease in the tumor growth rate (18d after inoculation) in comparison to PBS and PL-1 + anti-CD137 Ab (Supplementary Fig. 8d, e). However, no significant extension in the overall survival time was observed comparing PL1-CD137 + anti-CD137 Ab to PL-1 + anti-CD137 Ab treatment (Supplementary Fig. 8f). Here we show PL1 nanoparticle delivery of costimulatory receptor CD137 mRNA improved the immunotherapy with an anti-CD137 Ab to some extent in both tumor models with better results obtained in the B16F10 melanoma model as compared to the A20 lymphoma model.

### Regression of tumor growth with the treatment of PL1-OX40 mRNA + anti-OX40 antibody

The therapeutic effects of the costimulatory receptor OX40 delivery in the B16F10 melanoma and CT26 colon carcinoma models were explored. PL1-OX40 + anti-OX40 Ab treatment (i.t.) significantly decreased the tumor growth and prolonged survival in comparison to treatment with PBS and PL1-OX40 + anti-OX40 Ab in both tumor models (Supplementary Fig. 9a–f).

**Fig. 3:**
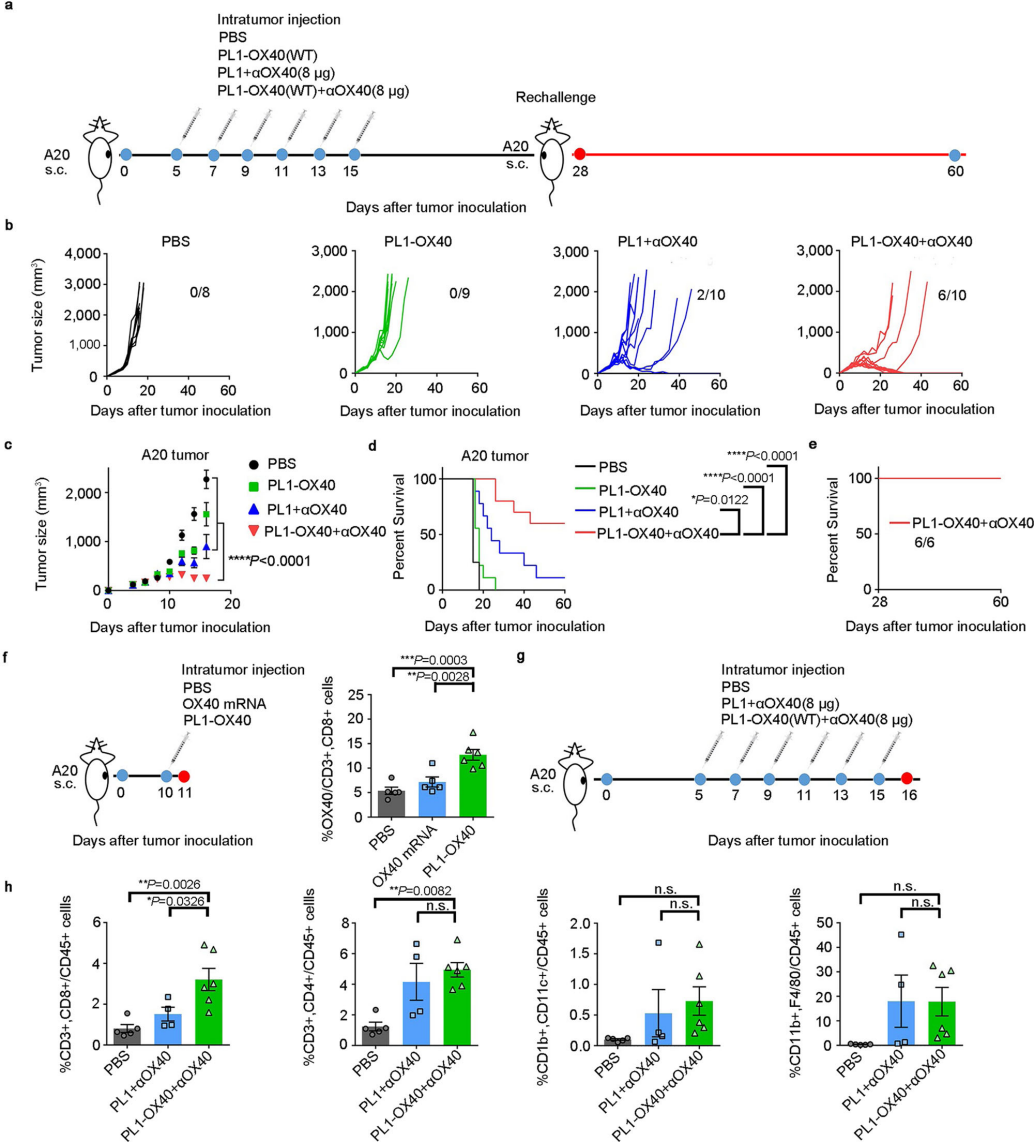
Regression of A20 tumors after treatment with PL1-OX40 + anti-OX40 antibody. **a** Schematic illustration of the A20 mouse tumor model and the treatment regimen. **b** Tumor volumes of individual mice (*n* = 8–10) after i.t. injected six doses of PBS, PL1-OX40, PL1 + anti-OX40 Ab, or PL1-OX40 + anti-OX40 Ab. PL1-OX40 (10 µg mRNA/mouse), and anti-OX40 Ab (8 µg/mouse). **c**, **d** Tumor volumes (**c**) and overall survival (**d**). *n* = 8 mice in PBS group, *n* = 9 mice in PL1-OX40 group, *n* = 10 mice in PL1 + anti-OX40 Ab group, and *n* = 10 mice in PL1-OX40 + anti-OX40 Ab group. **e** Overall survival of rechallenged mice. On day 28, rechallenge mice (*n* = 6) with complete response after the treatment with PL1-OX40 + anti-OX40 Ab. **f** Treatment plan for evaluation of OX40 expression on the CD8 + T cells after a single i.t. injection with PBS (*n* = 5), OX40 mRNA (*n* = 5), or PL1-OX40 (*n* = 6). **g**, **h** Immune cell analysis (CD8+, CD4+ T cells, macrophage, DC) after six i.t. injections with PBS (n = 5), PL1 + anti-OX40 Ab (*n* = 4), or PL1-OX40 + anti-OX40 Ab (*n* = 6), respectively. Data in **c**, **f**, and **h** are presented as the mean ± S.E.M. Statistical significance in **c** was analyzed with two-way ANOVA. Statistical significance in **d** was analyzed using the log-rank (Mantel-Cox) test. Statistical significance in **f** and **h** was analyzed using one-way ANOVA followed by Dunnett’s multiple comparison test. *P < 0.05; **P < 0.01; ***P < 0.001; ****P < 0.0001; n.s., not significant. Source data are provided as a Source Data file.

The therapeutic effects of PL1-OX40 + anti-OX40 Ab treatments were then evaluated in the A20 B cell lymphoma model. Mice received i.t. injections of PBS, PL1-OX40, PL1 + anti-OX40 Ab, or PL1-OX40 + anti-OX40 Ab. Tumor growth was monitored for 60 days (Fig. 3a, b). Treatment with PL1-OX40 + anti-OX40 Ab significantly reduced tumor growth (Fig. 3c) and increased the length of survival (Fig. 3d) when compared with the other three groups. Importantly, 6 out of 10 (60%) mice treated with PL1-OX40 + anti-OX40 Ab exhibited a complete response (Fig. 3d) and were resistant to A20 tumor rechallenge (Fig. 3e).

To study the role of mRNAs, we added two control groups, including OX40 mRNA + anti-OX40 Ab and PL1-OX40 (nocap) + anti-OX40 Ab. OX40 (nocap) mRNA has the same sequence as the OX40 mRNA but without a 5′ cap. The mRNA capping structures are known to protect transcripts from degradation during transcription and define the mRNA as “self” to avoid initiating innate immune responses. In the absence of the capping structure, mRNA translation efficiency is much lower, resulting in reduced OX40 expression^42^. We found that treatment with PL1-OX40 + anti-OX40 Ab significantly reduced tumor growth compared with OX40 mRNA + anti-OX40 Ab or PL1-OX40 (nocap) + anti-OX40 Ab controls (Supplementary Fig. 10a–c). These results indicated that PL1 nanoparticles delivering the costimulatory OX40 mRNA could enhance the immunotherapeutic effects of anti-OX40 Ab therapy in different mouse models.

Tumor-infiltrating lymphocytes (TIL) play an essential role in antitumor immunity^43,44^. mRNA delivery to TILs was explored in the A20 B cell lymphoma model. There was a significant increase in the expression of OX40 on tumor-infiltrating CD8+ T cells and dendritic cells (DCs) following PL1-OX40 treatment (Fig. 3f and Supplementary Fig. 11a), but there were minimal changes in the OX40 expression on infiltrating CD4+ T cells or macrophages (Supplementary Fig. 11a). Cytokine and chemokine levels were examined as well. Plasma levels of IFN-γ were significantly increased following PL1-OX40 treatment compared to control groups. Levels of the chemokine ligand 12 (CCL12), C-X-C motif chemokine ligand 1 (CXCL1), and macrophage colony-stimulating factor (M-CSF) were also increased by 2 to10-fold with the PL1-OX40 treatments (Supplementary Fig. 11b).

Infiltrating T-cell populations in the A20 B cell lymphoma tumors were then examined. Using the same dosing strategy as described in Fig. 3a, immune cell populations were analyzed 24 hrs following the last treatment (Fig. 3g). A significant increase in CD8+ T cells was observed, but there was no change in levels of CD4+ T cells, macrophages, or dendritic cells with the PL1-OX40 + anti-OX40 Ab treatment compared to the PL1 + anti-OX40 Ab (Fig. 3h). T-cell depletion (Supplementary Fig. 12) with either anti-CD8 or anti-CD4 Abs significantly compromised the efficacy of the combination treatment as compared to the administration of a control Ab. Next, we examined the effects of PL1-OX40 treatment on primary T cells. PL1-OX40 + anti-OX40 Ab treatment enhanced the secretion of TNF-α compared to the PBS control group, which indicated that T cells were activated (Supplementary Fig. 13a, b). Also, the delivery of PL1-OX40 in primary T cells induced higher T-cell proliferation compared to control groups (Supplementary Fig. 14).

In Supplementary Fig. 11a, we also noticed a significantly increased expression of OX40 on dendritic cells (DCs) following PL1-OX40 treatment. Recently, several studies reported that the OX40 and CD137 were also expressed on the DCs and were potentially beneficial to anti-cancer immunity^45,46^. To check whether the induced expression of OX40 on DCs contributed to the treatment outcome, we performed DCs depletion using anti-PDCA-1 Ab (Supplementary Fig. 15). Results showed that the DCs depletion significantly compromised the efficacy of the combination treatment of PL1-OX40 + anti-OX40 Ab as compared to the administration of a control Ab. We then investigated the expression of maturation and activation markers on primary DCs (including MHC II, CD80, CD40 and CD86) ex vivo after PL1-OX40 + anti-OX40 Ab treatment (Supplementary Fig. 16a–d). Results showed that CD80 expression was significantly higher in the PL1-OX40 + anti-OX40 Ab treatment group than the PBS group, indicating the current treatment strategy may activate DCs to some extent in the current immunotherapy.

Next, the cytokine and chemokine levels in mouse plasma after one-time treatment were analyzed by Luminex assays^18^. Several cytokines and chemokines, including regulated on activation normal T-cell expressed and secreted (RANTES/CCL5), macrophage inflammatory protein-1β (MIP-1B/CCL4), monokine induced by gamma interferon (MIG/CXCL9), monocyte chemoattractant protein-1 (MCP-1/CCL2), and interferon gamma-induced protein 10 (IP-10/CXCL10) were upregulated compared to that in the control groups (Supplementary Fig. 17), indicating the activation of anti-cancer immune responses. Additionally, after six doses of treatment, cytokines such as IFN-γ, CCL12, M-CSF, and CXCL1 in mouse plasma were similar in the different groups (Supplementary Fig. 18), which suggested no continuous cytokine inductions.

### Boosting antitumor efficacy of PL1-OX40 mRNA + anti-OX40 Antibody

**Fig. 4:**
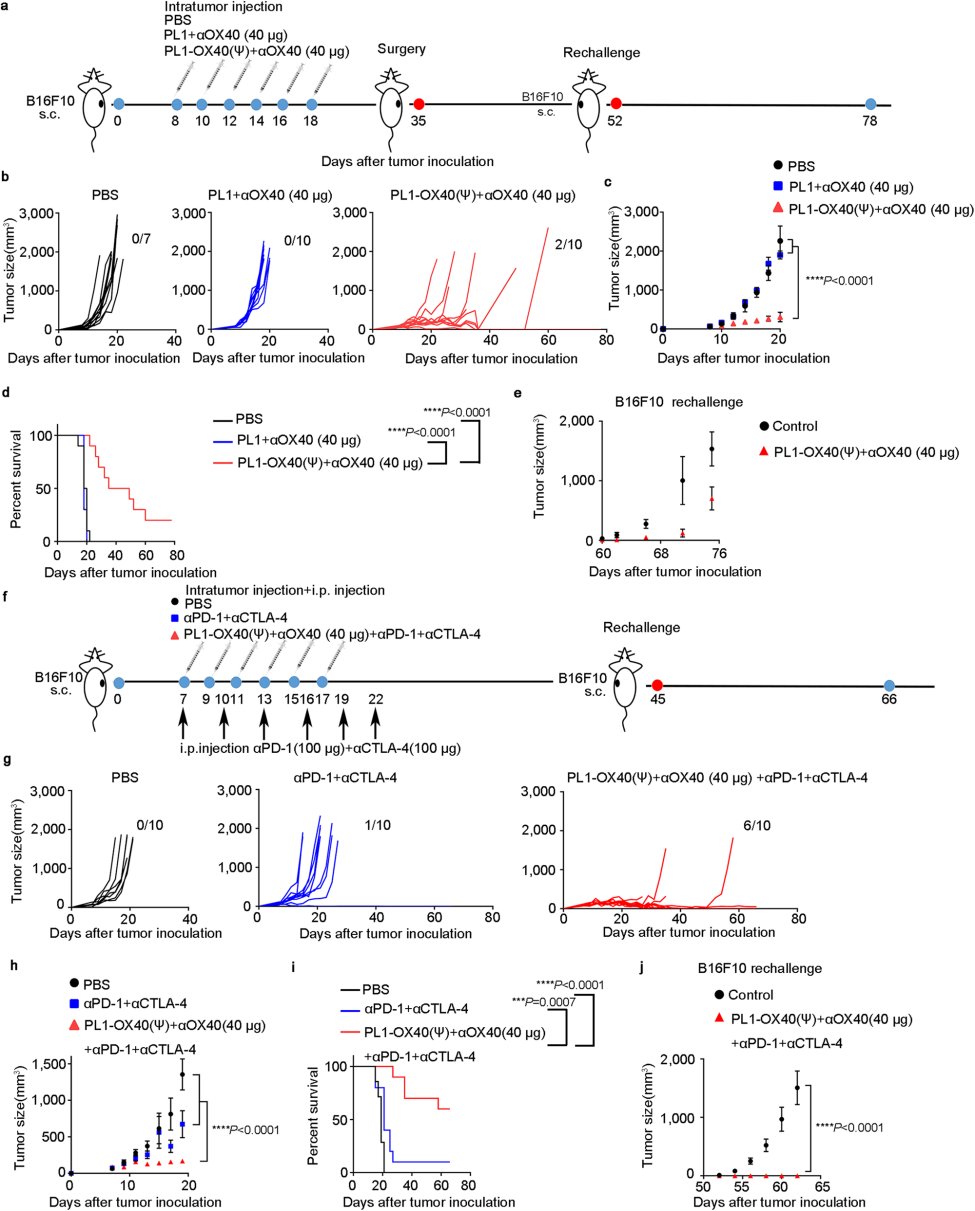
Antitumor efficacy of PL1-OX40 mRNA + anti-OX40 antibody when combined with surgery or checkpoint inhibitors. **a** Schematic illustration of the treatment of PL1-OX40 (ψ) mRNA + anti-OX40 (40 µg) Ab in combination with surgery (tumors volume <500 mm^3^). **b** Tumor volumes of individual mice (*n* = 7–10 per group) following six i.t. injections with PBS, PL1 + anti-OX40 (40 µg) or PL1-OX40 (ψ) + anti-OX40 Ab (40 µg). **c**, **d** Tumor volumes (**c**) and survival (**d**) of mice. *n* = 7 mice in PBS group, *n* = 10 mice in PL1 + anti-OX40 (40 µg) group, and *n* = 10 mice in PL1-OX40 (ψ) + anti-OX40 Ab (40 µg) group. **e** Tumor volumes of rechallenged tumor-free mice from **b** (*n* = 2) after surgery with native C57BL/6 mice as the control (*n* = 5). On day 78, the mice were removed. **f** Schematic illustration of the treatment of PL1-OX40 (ψ) mRNA + anti-OX40 (40 µg) Ab in combination with anti-PD-1 (100 µg) + anti-CTLA-4 (100 µg) Ab. Anti-PD-1 + anti-CTLA-4 Abs were injected intraperitoneally (i.p.) every 3 days for six doses. **g** Tumor volumes of individual mice received six doses of PBS (*n* = 10), anti-mouse PD-1 + anti-mouse CTLA-4 Abs (*n* = 10), or PL1-OX40 (ψ) + anti-OX40 (40 µg) Ab with anti-PD-1 Ab and anti-CTLA-4 Ab (*n* = 10) every other day. **h**, **i** Tumor volumes (**h**) and survival (**i**) of mice. *n* = 10 mice per group. **j** Tumor volumes of rechallenged mice that completely responded to the treatment with PL1-OX40 (ψ) + anti-OX40 (40 µg) + anti-PD-1 + anti-CTLA-4 antibodies (*n* = 6) vs. native control (*n* = 7). Data in **c**, **e**, **h**, and **j** are presented as the mean ± S.E.M. Statistical significance in **c**, **h**, and **j** were analyzed with two-way ANOVA. Statistical significance in **d** and **i** was analyzed with the log-rank (Mantel-Cox) test. ***P < 0.001; ****P < 0.0001. Source data are provided as a Source Data file.

Although the PL1-OX40 + anti-OX40 Ab treatments significantly decreased B16F10 tumor growth and prolonged survival (Supplementary Fig. 9a–c), complete eradication of the tumor burden is an important goal of immune-based treatments. To avoid exogenous mRNA triggering non-specific immune responses and enhance OX40 expression, OX40 mRNA was changed from its wild-type form (OX40 (WT)) to a pseudouridine (ψ)-modification form (OX40 (ψ)) (Supplementary Fig. 19)^47–49^. To further improve the antitumor effects of PL1-OX40 + anti-OX40 Ab therapy, the dosage of anti-OX40 antibody was increased from 8 to 40 µg per injection. Treatment of the B16F10 tumor-bearing mice with PL1-OX40 (ψ) + anti-OX40 Ab (40 µg) significantly decreased tumor growth and prolonged survival in comparison to PBS and PL1 + anti-OX40 treatment (Fig. 4a–d). At 35 days, five mice exhibited tumors with a size <500 mm^3^ in PL1-OX40 (ψ) + anti-OX40 Ab (40 µg) group, and surgery was performed to remove the tumors from these mice. At 52 d, two mice remained tumor-free. We rechallenged the surviving mice with B16F10 cells and used native C57BL/6 mice as controls. Compared to the control mice, the surviving mice showed delayed tumor growth with the rechallenge of B16F10 tumor cells (Fig. 4e).

In another treatment regimen, immune checkpoint inhibitors including anti-PD-1 + anti-CTLA-4 Abs were added to treatment with PL1-OX40 (ψ) + anti-OX40 Ab (40 µg) treatment (Fig. 4f). The combination of PL1-OX40 (ψ) + anti-OX40 with anti-PD-1 + anti-CTLA-4 Ab treatment dramatically inhibited tumor growth and prolonged survival in comparison to treatment with PBS or anti-PD-1 + anti-CTLA-4 Ab (Fig. 4g–i). At 45 days, six mice were tumor-free and one mouse had a small tumor (~50 mm^3^). The surviving mice were resistant to the rechallenged B16F10 tumor cells (Fig. 4j), among which the primary tumor of one mouse re-grew and met early removal criteria on 58 d. The remaining 5 mice remained tumor-free on both sides.

Also, we implanted B16F10 tumors at two different sites in the mice. PL1-OX40 (ψ) + anti-OX40 Ab was i.t. injected in only one tumor (Supplementary Fig. 20a). We then monitored tumor growth at both the injected and the distant sites. The results showed that PL1-OX40 (ψ) + anti-OX40 Ab enhanced antitumor effects of anti-PD-1 + anti-CTLA-4 Abs immunotherapy, inhibited tumor growth, and extended mouse survival (Supplementary Fig. 20b–e). Particularly, PL1-OX40 (ψ) + anti-OX40 Ab induced better antitumor effects of anti-CTLA-4 Ab immunotherapy than that of anti-PD-1 Ab.

**Fig. 5:**
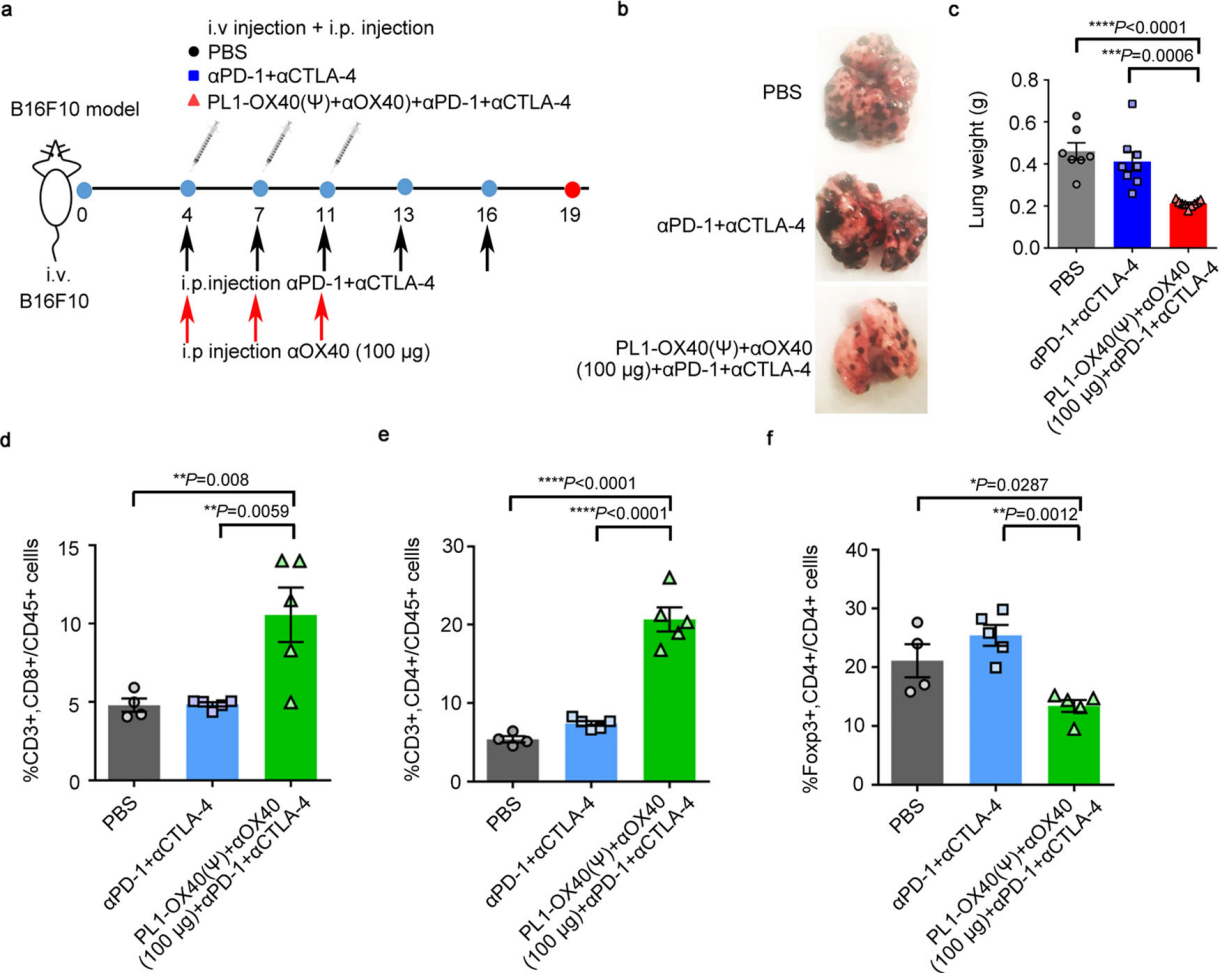
Antitumor efficacy in a lung metastasis mouse model. **a** Schematic illustration of lung metastases tumor of B16F10 cells with the treatment of PBS, anti-PD-1 + anti-CTLA-4 Abs, or PL1-OX40 mRNA + anti-OX40 Ab + anti-PD-1 + anti-CTLA-4 Abs. Mice received i.p. injections of PBS (*n* = 7), i.p. injections of anti-PD-1 (100 µg) + anti-CTLA-4 (100 µg) Abs (*n* = 8), or i.v. injections of PL1-OX40 (ψ) + i.p. injections of anti-OX40 (100 µg) Ab + anti-PD-1 (100 µg) Ab + anti-CTLA-4 (100 µg) Ab (*n* = 9) every 3 days as shown in the arrows. **b** Representative melanoma metastasis in the mouse lungs (Supplementary Fig. 22 for all lung samples in three groups). **c** Lung weight. **d**–**f** Immune cell analysis of CD8 + T cells, CD4 + T cells, Foxp3 + CD4 + (Treg) cells in the lungs from different treatments (*n* = 4, 5, 5), respectively. Data in **c**–**f** are presented as the mean ± S.E.M. Statistical significance in **c**–**f** was analyzed using one-way ANOVA followed by Dunnett’s multiple comparison test. *P < 0.05; **P < 0.01; ***P < 0.001; ****P < 0.0001. Source data are provided as a Source Data file.

Lastly, we checked the immunotherapy effects of the treatment regimen through systemic administrations. We first investigated the biodistribution of the PL1-mRNA in C57BL/6 mice via an i.v. injection of PL1-Fluc mRNA. After 24 h treatment, we found the PL1-Fluc mRNA was expressed mainly in the spleen and liver (Supplementary Fig. 21a). We also investigated the cell distribution of the PL1-GFP mRNA in mouse spleen. We found an increased expression of GFP in CD8+ T cells, macrophages, and DCs but not in CD4+ T cells (Supplementary Fig. 21b, c). Based on these results, we assessed the therapeutic efficacy of this treatment regimen in a B16F10 lung metastasis mouse model through systemic administrations of anti-PD-1 + anti-CTLA-4 Abs with PL1-OX40 (ψ) + anti-OX40 Ab (100 µg) (Fig. 5a). The results showed that this treatment regimen dramatically reduced the tumor metastasis in mouse lungs compared with anti-PD-1 + anti-CTLA-4 Abs and PBS treatment (Fig. 5b, c and Supplementary Fig. 22a, b). A significant increase of CD8+ and CD4+ T cells in mouse lungs was observed in the group of PL1-OX40 (ψ) + anti-OX40 Ab and anti-PD-1 + anti-CTLA-4 Abs compared to anti-PD-1 + anti-CTLA-4 Abs treatment (Fig. 5d, e and Supplementary Fig. 22c). Also, the number of Foxp3+ CD4+ cells (Treg cells) was decreased in the lungs in the group of PL1-OX40 (ψ) + anti-OX40 Ab with anti-PD-1 + anti-CTLA-4 Abs (Fig. 5f). These results indicate that systemic administrations of the treatment regimen demonstrate strong antitumor activity in the lung metastasis mouse model.

## Discussion

T-cell-based immunotherapy of cancer is a rapidly developing field^2^. Recently, nanotechnology has been developed to improve T-cell therapy^50,51^, such as ex vivo engineered T cells^52^ and in vivo modulation of T cells^53^. Despite these significant advances, one important challenge remains: to stimulate antitumor immunity of primary T cells in vivo. In this study, in order to explore nanoparticles for delivering mRNA into T cells, we designed and synthesized a library of phospholipid and glycolipid derivatives (PLs and GLs) and used these materials to formulate biomimetic nanoparticles for mRNA delivery. PL1 nanoparticles not only delivered the costimulatory receptor mRNA to a T-cell line in vitro, but were also able to deliver costimulatory receptor mRNA to T cells within the tumor in vivo, which provided a useful delivery material for the modulation of T-cell function.

Agonistic antibodies specific for costimulatory receptors with the ability to boost antitumor T-cell immunity have been developed for cancer treatment^27–29^. For example, the anti-OX40 antibody can activate T cells and enable them to eliminate tumor cells^31,53^. However, previous studies reported that low expression of OX40 hampered the anti-OX40 antibody immunotherapy effects in many tumor models (e.g., B16F10)^54^. In this study, PL1 nanoparticles were used to deliver OX40 mRNA into tumor-infiltrating T cells, which increased the expression of OX40 and consequently improved the antitumor effectiveness of anti-OX40 antibody. Combination treatment with PL1-OX40 and anti-OX40 antibody exhibited significant antitumor activity compared to the antibody alone in multiple tumor models. The composition of immune cells in the tumor microenvironment may contribute to the varied immunotherapeutic effects in different tumor models employed^55^. We observed an increased OX40 expression in CD8+ T cells in both A20 and B16F10 tumors. Although CD4+ T cells did not express OX40 in a significant level in A20 tumor, global CD4+ T-cell depletion impacts tumor growth through multiple mechanisms. We will continue to explore and untangle the antitumor mechanisms of this treatment in different tumor models. To further boost the antitumor activity of PL1-OX40 and anti-OX40 antibodies, we added anti-PD-1 and anti-CTLA-4 antibodies to the treatment regimen. This treatment approach resulted in an approximate 50% complete response in the B16F10 tumor model. Notably, these mice were resistant to B16F10 tumor rechallenge. These results indicate that this treatment regimen effectively induces antitumor immunity in vivo.

The TNF superfamily of receptors (TNFRSF) members CD137 and OX40 are well-known costimulatory receptors expressed on T cells that stimulate T-cell proliferation and activation upon interaction with their cognate ligands^27,33,34^. Some studies reported that the CD137 and OX40 were also expressed on the dendritic cells (DCs), which can bind to their agonistic antibodies, thereby enhancing the ability of DCs to stimulate T-cell proliferation and generate tumor antigen-specific CD8+ T-cell responses^45,46^. In this study, PL1-OX40 nanoparticles induced the OX40 expression on both T cells and DCs. The mechanisms of action studies showed that PL1-OX40 nanoparticles together with agonist OX40 antibody directly stimulated T cells. Meanwhile, this regimen activated DCs and triggered the subsequent stimulation of T cells.

Furthermore, we found that our treatment strategy is compatible with multiple administration routes. The combination treatment with PL1-OX40 and anti-OX40 antibody exhibited significant antitumor activity. More importantly, systemic administrations of anti-PD-1 + anti-CTLA-4 Abs with PL1-OX40 (ψ) + anti-OX40 Ab dramatically reduced the tumor metastasis in the lung metastasis model. These results demonstrate the broad applicability of this treatment regimen under diverse therapeutic situations. It is noteworthy that an increasing number of costimulatory receptors have been extensively investigated for their mechanism of actions such as CD28 or CD27, GITR, and ICOS^27^. Our results lay a solid foundation for the exploration of more effective combinations using costimulatory receptors and agonistic antibodies for cancer immunotherapy.

## Methods

### Antibodies

Antibodies were used for immunotherapy: anti-OX40 antibody (InVivoPlus anti-mouse OX40 (CD134), BioXcell, Cat: BP0031), anti-CD137 antibody (InVivoPlus anti-mouse 4-1BB (CD137), BioXcell, Cat: BP0169). Antibodies were used for FACS:anti-CD45 APC (30-F11) (Thermo Fisher, Cat: # 17-0451-82), anti-CD3 PE (145-2C11) (BD Pharmingen, Cat: # 553063), anti-CD4 Pacific Blue (RM4-5) (Thermo Fisher Cat: # MCD0428), anti-CD8 APC-efluor 780 (53-4.7) (Thermo Fisher, Cat: # 47-0081-82), anti-CD11b Pacific Blue (M1/70.15) (Thermo Fisher, Cat: # RM2828), anti-F4/80 PE-eFluor 610 (BM8) (Thermo Fisher, Cat: # 61-4801-82), anti-CD11c PE-Cyanine7 (N418) (Thermo Fisher, Cat: # 25-0114-82), anti-Foxp3-PE-eFluor® 610 (FJK-16s) (Thermo Fisher, Catalog # 61-5773-82), anti-CD137-FITC (1AH2) (BD Pharmingen, Catalog No. 558975) and anti-OX40 FITC (OX86) (Abcam, Cat: # ab33998). Antibodies were used for immunofluorescence image: anti-CD8 PE-Texas red (Abcam, Cat:# ab25294), anti-CD4 Alexa488 (BD biosciences, Cat: # 557667).

### Cell lines

Mouse cell lines E.G7 (E.G7-OVA, ATCC-2113), A20 (TIB-208), and CT26 (CRL-2638) were purchased from American Type Culture Collection (ATCC). Cells were cultured according to the instructions from ATCC. The mouse cell line B16F10 was obtained from the lab of Dr. Jianhua Yu. Cells were cultured with Dulbecco’s modified Eagle medium (Corning) containing 10% fetal bovine serum (Invitrogen) in an incubator at 37 °C in 5% CO_2_.

### Animals

All animal studies were approved by the Institutional Animal Care and Use Committee (IACUC) at Ohio State under protocols 2014A00000106-R2. The studies were carried out in compliance with all relevant ethical regulations. Female BALB/c and C57BL/6 mice (6–8 weeks) were purchased from the Jackson Laboratory, and housed in single-unit cages with 12-h alternate light and dark cycles and at controlled ambient temperature (68–79 °F) at the university laboratory animal resources-Biomedical Research Tower of The Ohio State University.

### mRNA synthesis by in vitro transcription (IVT)

The linear dsDNA sequences of firefly luciferase, GFP, mouse OX40, and mouse CD137 were purchased from Integrated DNA Technologies (IDT), and inserted into a pUC19 vector via the Golden Gate assembly. The plasmids were amplified to generate the DNA template for in vitro transcription. Then, mRNAs of luciferase, GFP, mouse OX40, and mouse CD137 were prepared through in vitro transcription (AmpliScribe™ T7-Flash™ Transcription Kit, Lucigen) followed by purification using RNA Clean & Concentrator (Zymo). A cap-1 structure was then installed for the mRNAs (Vaccinia Capping System, NEB; Cap 2′-O-methyltransferase, NEB) followed by another round of RNA purification (Zymo). The concentration of the synthesized mRNA was measured by a NanoDrop 2000 Spectrophotometer (Thermo), and the mRNA was then stored at −80°C for future use.

### Preparation and characterization of PLs and GLs nanoparticles

Briefly, the PLs and GLs nanoparticles were formulated (PLs or GLs: DOPE: Cholesterol: DMG-PEG2000 = 20: 30: 40: 0.75 molar ratio) with FLuc/GFP/OX40/CD137 mRNA (lipid/mRNA = 10/1, mass ratio). PLs or GLs, DOPE, Cholesterol, and DMG-PEG2000 were dissolved and mixed in ethanol, and the mRNAs were dissolved in 10 mM citric acid (pH = 3). The nanoparticles were prepared as 1:3 of the above solutions by pipetting or by a microfluidic device (Precision NanoSystems, Vancouver, BC, Canada) for in vitro or in vivo experiments, respectively^39–41^. These nanoparticles were dialyzed in 1X PBS for 80 min before treating animals. The size and zeta potential of nanoparticles were measured by NanoZS Zetasizer (Malvern, Westborough, MA, USA). mRNA encapsulation efficiency (EE%) was calculated as 1-(sample without Triton® X-100/sample with Triton® X-100) via a RiboGreen assay. The morphology of nanoparticles was examined on a Thermo Scientific™ Glacios™ cryo-TEM. Briefly, cryo-TEM samples were prepared by transfering 3 μL of PL-1 mRNA NPs to a specimen grid. After removing excess liquid, the grid was immediately plunged into liquid ethane to rapidly form a thin film of amorphous ice using Vitrobot Mark IV system (Thermo Fisher Scientific, Hillsboro). Next, the Cryo-TEM images were collected at a nominal magnification of 57,000× with Falcon direct electron detector on Thermo Scientific™ Glacios™ CryoTEM. The microscope was operated at an acceleration voltage of 200 kV.2 × 10^5^ E.G7-OVA cells were seeded into a 96-well plate. After 6 h, cells were treated with nanoparticles encapsulating Fluc mRNA for 18 h. The delivery efficiency of mRNA was determined by the luciferase expression assay^39^.

### GFP, CD137, and OX40 expression in vitro

In all, 2 × 10^5^ E.G7-OVA cells were seeded into a 24-well plate. After 6 h, cells were treated with PBS, 400 ng free GFP/OX40/CD137 mRNA, or 400 ng PL1-GFP/PL1-OX40/PL1-CD137 mRNA for 18 h. After washed with PBS, cells were stained with the anti-OX40-FITC (OX86) (Abcam, Cat: ab33998, 1/50) or anti-CD137-FITC (1AH2) (BD Pharmingen, Cat: 558975, 1/50) antibodies. Then, cells were washed with PBS three times and analyzed on a BD LSR Fortessa or BD LSR II flow cytometer.

### Endocytic pathways of PL1 nanoparticles

In all, 1 × 10^5^ E.G7-OVA cells were seeded into each well of 24-well plates in 500 µL growth medium for 24 h at 37 °C with 5% CO_2_. The cells were treated with 5-(*N*-Methyl-*N*-isopropyl)amiloride, chlorpromazine hydrochloride, or methyl-β-cyclodextrin at the concentrations of 20 μM, 10 µM, and 4.5 mM, respectively. After 0.5 h treatment, each well was treated with 250 ng PL1 nanoparticles encapsulating Alexa-Fluor 647-labeled RNA. After 3 h culture, the cells were washed and evaluated via flow cytometry analysis (BD Biosciences).

### GFP, CD137, and OX40 expression in tumor models

Tumor cells (1 × 10^5^ B16F10 or 5 × 10^6^ A20) were injected subcutaneously on the right flank of C57BL/6 or BALB/c mice. Mice with tumor size ~0.8 to 0.9 cm in the largest diameter were randomized to different treatment groups. For one-time injection, mice were i.t. treated with PBS/free mRNA (20 μg) or PL1-GFP/OX40/CD137 mRNA (20 μg mRNA). For multiple injections, mice were i.t. treated with PBS, PL1 + anti-OX40 Ab, or PL1-OX40 + anti-OX40 Ab for six doses. After 24 h, the tumors were mechanically dissociated using a MACS dissociator and gently digested via a Tumor Dissociation Kit, Mouse (MACS, 130-096-730) according to the protocol. Cells were stained with the antibody combination of CD45 APC, CD3 PE, CD4 Pacific Blue, CD8 APC-efluor 780 and OX40/CD137 FITC, CD45 APC, CD11b Pacific Blue, F4/80 PE-eFluor 610, CD11c PE-Cyanine7 and OX40/CD137 FITC. anti-CD45 APC (30-F11) (Thermo Fisher, Cat:17-0451-82, 1/200), anti-CD3 PE (145-2C11) (BD Pharmingen, Cat: 553063, 1/200), anti-CD4 Pacific Blue (RM4-5) (Thermo Fisher Cat:MCD0428, 1/50), anti-CD8 APC-efluor 780 (53-4.7) (Thermo Fisher, Cat:47-0081-82, 1/200), or the combination of anti-CD45 APC (30-F11) (Thermo Fisher, Cat:17-0451-82, 1/200), anti-CD11b Pacific Blue (M1/70.15) (Thermo Fisher, Cat: RM2828, 1/50), anti-F4/80 PE-eFluor 610 (BM8) (Thermo Fisher, Cat: 61-4801-82, 1/200), anti-CD11c PE-Cyanine7 (N418) (Thermo Fisher, Cat: 25-0114-82, 1/200), anti-OX40 FITC (OX86) (Abcam, Cat: # ab33998, 1/100), and anti-CD137-FITC (1AH2) (BD Pharmingen, Catalog No. 558975, 1/100). GFP/OX40/CD137 expressions in TIL population (Supplementary Fig. 23) were quantified by FACS on a BD LSR Fortessa or BD LSR II flow cytometer (BD Biosciences).

### Tumor models treated with PL1-OX40/CD137 and the corresponding agonist Ab

Tumor cells (5 × 10^6^ A20, 5 × 10^5^ CT26, or 1 × 10^5^ B16F10) were injected subcutaneously on the right flank of BALB/c or C57BL/6 mice. Mice with tumor size ~0.5 to 0.7 cm in the largest diameter were randomized to different treatment groups: PBS, free PL1 + Ab, and PL1-OX40/CD137 mRNA + Ab. PBS, free PL1, or PL1- OX40/CD137 mRNA (10 μg mRNA/mouse) was injected directly into the tumors. After 4 h, mice were i.t. injected with PBS or Ab (anti-OX40 8 μg, anti-CD137 16 μg). The injections were performed every other day for six doses. Tumor size was monitored with a digital caliper every 2 to 3 days and calculated as the volume (length × width × width/2). Mice were sacrificed when tumor size reached 1.6 cm in diameter.

### A20 tumor models with rechallenge

A20 tumor model was established and treated with PBS, PL1-OX40, PL1 + anti-OX40 Ab, or PL1-OX40 (10 μg/mouse) + anti-OX40 Ab every other day for six doses. Twenty-eight days after the tumor inoculation, survived mice from the PL1-OX40 + anti-OX40 Ab group were rechallenged with 5 × 10^6^ A20 cells on the other side (*n* = 6). Tumor size on both sides were monitored with a digital caliper every 2 to 3 days and calculated as the volume (length × width × width/2). Mice were sacrificed when tumor size reached 1.6 cm in diameter.

### T cells and DCs depletion in A20 tumor model treated with PL1-OX40 + anti-OX40 Ab

Mice were divided into different groups (*n* = 9 or 10 per group): isotype control antibody + PL1-OX40 + anti-OX40 Ab, anti-CD8 + PL1-OX40 + anti-OX40 Ab, anti-CD4+ PL1-OX40 + anti-OX40 Ab, or anti- PDCA-1 + PL1-OX40 + anti-OX40 Ab. PL1-OX40 (10 µg mRNA/mouse) + anti-OX40 (8 µg) Ab were i.t. injected every other day for six doses. Anti-mouse CD8α (α-CD8, Bioxcell, BE0004-1, 200 μg per mouse, i.p.), anti-mouse CD4 (α-CD4, Bioxcell, clone BP0003-1, 200 μg per mouse, i.p.), anti-mouse PDCA-1 (αPDCA-1, Bioxcell, clone: 927, the first dose is 500 μg per mouse, second and third doses is 250 μg per mouse, i.p.) or anti-rat IgG2b isotype control (Bioxcell, Catalog # BP0090, 200 μg per mouse, i.p.) were given every 3 days for three doses starting from 1 day before PL1-OX40 and anti-OX40 Ab treatment.

### Luminex analysis and ELISA of plasma cytokines and chemokines in A20 tumor model

Mice were i.t. treated once as mentioned in the text. 24 h after treatment, blood was collected by cardiac puncture with sodium citrate (final concentration 1%, w/v) in a syringe. After centrifuging the whole blood at 1,500 g for 30 min at 4 °C, the plasma in the supernatant was collected and stored at −80 °C. Mouse blood plasma concentration of IFN-gamma (Catalog#, MIF00), CXCL1/KC (Catalog#, MKC00B), M-CSF (Catalog#, MMC00), and CCL12/MCP-5 (Catalog#, MCC120) were tested by quantitative ELISA kits (R&D Systems, Inc.). The total plasma protein was quantified by Pierce™ BCA Protein Assay Kit following the manufacturer’s instructions. Mouse cytokines and chemokines levels in plasma were determined by Luminex assay (Eve Technologies, Canada). Row data were normalized by calculating the standard score (Z-score). Z-score = (protein level in each sample-row mean)/row standard deviation (SD). Mouse plasma concentrations of IFN-gamma, CXCL1/KC, M-CSF, and CCL12/MCP-5 were quantified by quantitative ELISA kits.

### Combination of PL1-OX40 + anti-OX40 Ab treatment with surgical removal

In all, 1 × 10^5^ B16F10 tumor cells were injected subcutaneously in C57BL/6 mice. Mice were randomized to three groups: PBS, PL1 + anti-OX40 (40 μg), and PL1-OX40 (ψ) + anti-OX40 (40 μg). PL1-OX40 (ψ) (10 µg mRNA/mouse) were i.t. injected, and after 4 h, anti-OX40 Ab (40 µg/mouse) were i.t. injected. Six i.t. doses were given every other day. For the combination therapy with surgery, at d 35, five mice with tumors (<500 mm^3^) were removed with surgery. At d 52, two mice with undetectable tumor were rechallenged with B16F10 cells (1 × 10^5^) with native C57BL/6 mice as the control. For the combination therapy with anti-PD-1 + anti-CTLA-4 antibodies, anti-mouse PD-1 (100 µg/mouse, clone: RMP1-14, BioXcell) and anti-mouse CTLA-4 (100 µg/mouse, clone: 9D9, BioXcell) antibodies were i.p. injected every 3 day for six doses. At day 45, six mice with undetectable tumor were rechallenged with 1 × 10^5^ B16F10 cells with native C57BL/6 mice as the control.

### Combination of PL1-OX40 + anti-OX40 Ab treatment with checkpoint inhibitors in a lung metastasis model

A B16F10 lung metastasis mouse model was established by an intravenous injection of 2 × 10^5^ B16F10 cells into C57BL/6 mice. Mice received five doses of i.p. injections of PBS (*n* = 7), five doses of i.p. injections of anti-mouse PD-1 + anti-mouse CTLA-4 Abs (*n* = 8), or three doses of i.v. injections of PL1-OX40 (ψ) + i.p. injections of anti-OX40 Ab (100 µg) + five doses of i.p. injections of anti-PD-1 Ab + anti-CTLA-4 Ab (*n* = 9) every 3 days. Nineteen days after i.v. injection of B16F10 cells, mice were sacrificed and analyzed for cell and organ characteristics. Mouse lungs were imaged and weighed. Cell populations (CD8+, CD4+ T cells, Foxp3+CD4+ (Treg) cells) in mouse lungs from different treatments (*n* = 4, 5, 5) were analyzed by a BD LSR Fortessa or BD LSR II flow cytometer (BD Biosciences). The remaining mice from each group were used for immunofluorescence staining with different antibodies: CD4 Alexa488 (BD biosciences, Cat: # 557667) and CD8 PE-Texas red (Abcam, Cat:# ab25294) following the manufacturer’s instructions. The slides were imaged with a confocal microscope (Nikon A1R).

### Characterizations of primary T cells ex vivo with the treatment of PL1-OX40 and anti-OX40 antibody

CD8 and CD4 T cells were isolated from mouse spleen (T-cell isolation kits were ordered from Miltenyi Biotec). After 24-h activation by CD3ε and CD28 beads (Mouse T cell Activation and Expansion Kit, Miltenyi Biotec #130-093-627), the cell suspension was diluted to 2 × 10^5^ T cells/mL (50% CD4 and 50% CD8 T cells). In all, 100 μL cell suspension (2 × 10^4^ T cells) was added into each well of a 96-well plate. The T cells were treated by PBS or 5 μL (0.01 mg/mL) PL1-OX40 (WT) or PL1-OX40 (ψ) for 12 h. Then, CD3ε and CD28 beads were removed. Lastly, the T cells were stained with OX40-FITC antibody and then detected by flow cytometry. Similarly, T-cell proliferation and activation were performed, respectively. The T cells were labeled by CellTrace™ CFSE (CellTrace™ Cell Proliferation Kits, CellTrace™ CFSE, C34570) and were resuspended in the medium containing CD3 beads and 5 μg/mL anti-OX40 Ab. After 60 h, the cell number was analyzed by a flow cytometer with a 488 nm excitation source. Additionally, intracellular IFN-γ and TNF-α were quantified by ELISA.

### Characterizations of bone marrow derived dendritic cells (BMDCs) ex vivo with the treatment of PL1-OX40 and anti-OX40 antibody

Monocytes were isolated from mouse bone marrow. After 8-day culture with 50 ng/mL GMCSF and 50 ng/mL IL4, bone marrow derived dendritic cells were purified with CD11c beads (Miltenyi Biotec, Order no. 130-108-338). BMDCs were treated with PL1-OX40 mRNA (50 ng mRNA/2 × 10^4^ cells). After 12-h treatment, BMDCs were cultured with OX-40 Ab (5 μg/mL) for additional 12 h. The cells were stained with MHC II, CD80, CD86, and CD40 antibodies and then detected by flow cytometry.

### A20 Tumor models treated with free OX40 mRNA + anti-OX40 Ab, PL1-OX40 (nocap) + anti-OX40 Ab or PL1-OX40 + anti-OX40 Ab

Tumor cells (5 × 10^6^ A20) were injected subcutaneously on the right flank of BALB/c mice. Mice with tumor size ~0.5 to 0.7 cm in the largest diameter were randomized to different treatment groups: PBS, free OX40 mRNA + anti-OX40 Ab, PL1-OX40 mRNA (nocap) + anti-OX40 Ab, and PL1-OX40 mRNA + anti-OX40 Ab. OX40 (nocap) with the same sequence with OX40 mRNA was produced without the process of 5′ capping process on the OX40 mRNA. PBS, free OX40 mRNA, PL1- OX40 (nocap) mRNA or PL1- OX40 mRNA (10 μg mRNA/mouse) was injected directly into the tumors. After 4 h, mice were i.t. injected with PBS or Ab (anti-OX40 8 μg). The injections were performed every other day for six doses. Tumor size was monitored with a digital caliper every 2 to 3 days and calculated as the volume (length × width × width/2). Mice were sacrificed when tumor size reached 1.6 cm in diameter.

### Biodistribution of PL1-Fluc luciferase mRNA in vivo

C57BL/6 mice were implanted s.c. with B16F10 melanoma cells. The mice were i.t. injected with PL1-Fluc (20 µg mRNA/mouse). In a separate experiment, C57BL/6 mice were i.v. injected with B16F10 melanoma cells. After 3 days, the mice were i.v. injected with PL1-Fluc (20 µg mRNA/mouse). After 24 h, the mice were injected with luciferin intraperitoneally and imaged by a Xenogen IVIS spectrum in vivo imaging system (Caliper).

### Biodistribution of PL1-GFP mRNA in spleen cells in vivo

C57BL/6 mice were i.v. injected with PL1-GFP (20 µg mRNA/mouse). After 24 h, the mice were sacrificed. The spleen was mechanically dissociated using a MACS dissociator and gently digested via a Spleen Dissociation Kit, mouse (MACS, 130-095-926) according to the protocol. Cells were stained with the antibody combination of CD45 APC, CD3 PE, CD4 Pacific Blue, CD8 APC-efluor 780 and CD45 APC, CD11b Pacific Blue, F4/80 PE-eFluor 610, CD11c PE-Cyanine7. GFP expression in lymphocytes of spleen were quantified by FACS on a BD LSR Fortessa or BD LSR II flow cytometer (BD Biosciences).

### Two-side tumor model of B16F10 treated with PL1-OX40 + anti-OX40 Ab combined with anti-PD-1 + anti-CTLA-4 Abs

In all, 1 × 10^5^ B16F10 tumor cells were injected subcutaneously on the right flank of C57BL/6 as treated tumor. After 4 days 1 × 10^5^ B16F10 tumor cells were injected subcutaneously on the left flank of C57BL/6 as untreated tumor. Mice were randomized to different groups: PBS, anti-PD-1 +  anti-CTLA-4, PL1-OX40(ψ) + anti-OX40 (40 μg) +  anti-PD-1, PL1-OX40(ψ) + anti-OX40 (40 μg) + anti-CTLA-4, and PL1-OX40(ψ) + anti-OX40 (40 μg) + anti-PD-1 + anti-CTLA-4. PL1-OX40 mRNA (10 μg/mouse) was i.t. injected. After 4 h, tumors were i.t. injected with anti-OX40 Ab (40 μg/mouse) every other day for six doses. anti-PD-1 and/or anti-CTLA-4 (100 μg/mouse) were i.p injected every 3 days for six doses.

### Statistics

Statistical analyses were conducted on GraphPad Prism 7. Two-way ANOVA with repeated measurements was conducted on R3.4.3 (www.r-project.org). Tumor volumes: Two-way ANOVA with repeated measurements. Survival: Log-rank test. All Student’s *t*-tests were two-tailed. One-way ANOVA followed by Dunnett’s multiple comparison test was used to compare multiple experimental groups with the control (positive or negative). The *p* values were shown in the figures and specific statistical methods were described in the figure legends. *P < 0.05, **P < 0.01, ***P < 0.001, ****P < 0.0001 was considered statistically significant.

### Reporting summary

Further information on research design is available in the Nature Research Reporting Summary linked to this article.

## Supplementary information

Supplementary Information

Reporting Summary

## Data Availability

Source data are provided with this paper. The synthesis routes and analytical information of the chemical derivatives presented in Fig. 1 are provided in the Supplementary Information document. The remaining information are available within the Article, Supplementary Information or Source Data file. Source data are provided with this paper.

## Source data

Source Data

## References

[1] O’Donnell JS, Teng MWL, Smyth MJ (2019). Cancer immunoediting and resistance to T cell-based immunotherapy. Nat. Rev. Clin. Oncol..

[2] Sanmamed MF, Chen L (2018). A paradigm shift in cancer immunotherapy: from enhancement to normalization. Cell.

[3] Riley RS, June CH, Langer R, Mitchell MJ (2019). Delivery technologies for cancer immunotherapy. Nat. Rev. Drug Discov..

[4] Scheetz L (2019). Engineering patient-specific cancer immunotherapies. Nat. Biomed. Eng..

[5] Goldberg MS (2019). Improving cancer immunotherapy through nanotechnology. Nat. Rev. Cancer.

[6] Hu Z, Ott PA, Wu CJ (2018). Towards personalized, tumour-specific, therapeutic vaccines for cancer. Nat. Rev. Immunol..

[7] Luo M (2017). A STING-activating nanovaccine for cancer immunotherapy. Nat. Nanotechnol..

[8] Kuai R, Ochyl LJ, Bahjat KS, Schwendeman A, Moon JJ (2017). Designer vaccine nanodiscs for personalized cancer immunotherapy. Nat. Mater..

[9] Stadtmauer, E. A. et al. CRISPR-engineered T cells in patients with refractory cancer. *Science* **367**, eaba7365 (2020). 10.1126/science.aba7365 PMC11249135 32029687

[10] Rafiq S, Hackett CS, Brentjens RJ (2020). Engineering strategies to overcome the current roadblocks in CAR T cell therapy. Nat. Rev. Clin. Oncol..

[11] Ma L (2019). Enhanced CAR-T cell activity against solid tumors by vaccine boosting through the chimeric receptor. Science.

[12] Hu Q (2018). Conjugation of haematopoietic stem cells and platelets decorated with anti-PD-1 antibodies augments anti-leukaemia efficacy. Nat. Biomed. Eng..

[13] Chambers CA, Kuhns MS, Egen JG, Allison JP (2001). CTLA-4-mediated inhibition in regulation of T cell responses: mechanisms and manipulation in tumor immunotherapy. Annu. Rev. Immunol..

[14] Ishida Y, Agata Y, Shibahara K, Honjo T (1992). Induced expression of PD-1, a novel member of the immunoglobulin gene superfamily, upon programmed cell death. EMBO J..

[15] Wang, C., Ye, Y., Hu, Q., Bellotti, A. & Gu, Z. Tailoring biomaterials for cancer immunotherapy: emerging trends and future outlook. *Adv. Mater*. **29**, 201606036 (2017). 10.1002/adma.201606036 28556553

[16] Sagiv-Barfi, I. et al. Eradication of spontaneous malignancy by local immunotherapy. *Sci. Transl. Med.* **10**, eaan4488 (2018). 10.1126/scitranslmed.aan4488 PMC5997264 29386357

[17] Chester C, Sanmamed MF, Wang J, Melero I (2018). Immunotherapy targeting 4-1BB: mechanistic rationale, clinical results, and future strategies. Blood.

[18] Haabeth OAW (2019). Local delivery of Ox40l, Cd80, and Cd86 mRNA kindles global anticancer immunity. Cancer Res..

[19] Miao L (2019). Delivery of mRNA vaccines with heterocyclic lipids increases anti-tumor efficacy by STING-mediated immune cell activation. Nat. Biotechnol..

[20] Islam MA (2018). Restoration of tumour-growth suppression in vivo via systemic nanoparticle-mediated delivery of PTEN mRNA. Nat. Biomed. Eng..

[21] Wang H, Mooney DJ (2018). Biomaterial-assisted targeted modulation of immune cells in cancer treatment. Nat. Mater..

[22] Gosselin EA, Eppler HB, Bromberg JS, Jewell CM (2018). Designing natural and synthetic immune tissues. Nat. Mater..

[23] Zhuang J (2019). Nanoparticle delivery of immunostimulatory agents for cancer immunotherapy. Theranostics.

[24] Kroll, A. V. et al. Nanoparticulate delivery of cancer cell membrane elicits multiantigenic antitumor immunity. *Adv. Mater.* **29**, 201703969 (2017). 10.1002/adma.201703969 PMC5794340 29239517

[25] Minn AJ, Wherry EJ (2016). Combination cancer therapies with immune checkpoint blockade: convergence on interferon signaling. Cell.

[26] Vonderheide RH (2018). The immune revolution: a case for priming, not checkpoint. Cancer Cell.

[27] Driessens G, Kline J, Gajewski TF (2009). Costimulatory and coinhibitory receptors in anti-tumor immunity. Immunol. Rev..

[28] Melero I (1997). Monoclonal antibodies against the 4-1BB T-cell activation molecule eradicate established tumors. Nat. Med..

[29] Weinberg AD (2000). Engagement of the OX-40 receptor in vivo enhances antitumor immunity. J. Immunol..

[30] Mahoney KM, Rennert PD, Freeman GJ (2015). Combination cancer immunotherapy and new immunomodulatory targets. Nat. Rev. Drug Discov..

[31] Ma BY (2005). The expression and the regulatory role of OX40 and 4-1BB heterodimer in activated human T cells. Blood.

[32] Aspeslagh S (2016). Rationale for anti-OX40 cancer immunotherapy. Eur. J. Cancer.

[33] Taraban VY (2002). Expression and costimulatory effects of the TNF receptor superfamily members CD134 (OX40) and CD137 (4-1BB), and their role in the generation of anti-tumor immune responses. Eur. J. Immunol..

[34] Buchan SL, Rogel A, Al-Shamkhani A (2018). The immunobiology of CD27 and OX40 and their potential as targets for cancer immunotherapy. Blood.

[35] Marin-Acevedo JA (2018). Next generation of immune checkpoint therapy in cancer: new developments and challenges. J. Hematol. Oncol..

[36] Cooper GM (2000). The Cell: A Molecular Approach.

[37] Anderson MA, Shim H, Raushel FM, Cleland WW (2001). Hydrolysis of phosphotriesters: determination of transition states in parallel reactions by heavy-atom isotope effects. J. Am. Chem. Soc..

[38] Balaji BS, Lewis MR (2009). Double exponential growth of aliphatic polyamide dendrimers via AB(2) hypermonomer strategy. Chem. Commun. (Camb).

[39] Li B (2015). An orthogonal array optimization of lipid-like nanoparticles for mRNA delivery in vivo. Nano Lett..

[40] Zhang X (2017). Biodegradable amino-ester nanomaterials for Cas9 mRNA delivery in vitro and in vivo. ACS Appl. Mater. Interfaces.

[41] Zhang C (2019). Chemotherapy drugs derived nanoparticles encapsulating mRNA encoding tumor suppressor proteins to treat triple-negative breast cancer. Nano Res..

[42] Galloway A, Cowling VH (2019). mRNA cap regulation in mammalian cell function and fate. Biochim. Biophys. Acta Gene Regul. Mech..

[43] Stanton SE, Disis ML (2016). Clinical significance of tumor-infiltrating lymphocytes in breast cancer. J. Immunother. Cancer.

[44] Oble DA, Loewe R, Yu P, Mihm MC (2009). Focus on TILs: prognostic significance of tumor infiltrating lymphocytes in human melanoma. Cancer Immun..

[45] Wilcox RA (2002). Cutting edge: expression of functional CD137 receptor by dendritic cells. J. Immunol..

[46] Poropatich K (2020). OX40+ plasmacytoid dendritic cells in the tumor microenvironment promote antitumor immunity. J. Clin. Invest..

[47] Song JH, Yi CQ (2017). Chemical modifications to RNA: a new layer of gene expression regulation. Acs Chem. Biol..

[48] Li B, Luo X, Dong YZ (2016). Effects of chemically modified messenger RNA on protein expression. Bioconjugate Chem..

[49] Li, B. et al. Engineering CRISPR-Cpf1 crRNAs and mRNAs to maximize genome editing efficiency. *Nat. Biomed. Eng.* **1**, 0066 (2017). 10.1038/s41551-017-0066 PMC5562407 28840077

[50] Smith TT (2017). In situ programming of leukaemia-specific T cells using synthetic DNA nanocarriers. Nat. Nanotechnol..

[51] Lokugamage, M. P., Sago, C. D., Can, Z. B., Krupczak, B. R. & Dahlman, J. E. Constrained nanoparticles deliver siRNA and sgRNA to T cells in vivo without targeting ligands. *Adv. Mater.* **31**, 201902251 (2019). 10.1002/adma.201902251 PMC6819129 31465135

[52] Tang L (2018). Enhancing T cell therapy through TCR-signaling-responsive nanoparticle drug delivery. Nat. Biotechnol..

[53] Mayes PA, Hance KW, Hoos A (2018). The promise and challenges of immune agonist antibody development in cancer. Nat. Rev. Drug Discov..

[54] Yu, J. W. et al. Tumor-immune profiling of murine syngeneic tumor models as a framework to guide mechanistic studies and predict therapy response in distinct tumor microenvironments. *PLoS ONE* **13**, e0206223 (2018). 10.1371/journal.pone.0206223 PMC6214511 30388137

[55] Chen Z (2018). seq-ImmuCC: cell-centric view of tissue transcriptome measuring cellular compositions of immune microenvironment from mouse RNA-seq data. Front. Immunol..

