# Supplementary material for: Biomimetic nanoparticles deliver mRNAs encoding costimulatory receptors and enhance T cell mediated cancer immunotherapy
Source: Nat Commun. 2021 Dec 14;12:7264. doi: 10.1038/s41467-021-27434-x (PMC8671507; doi:10.1038/s41467-021-27434-x)
Supplement: Supplementary file 1 — Supplementary Information [file 41467_2021_27434_MOESM1_ESM.pdf]

Supplementary Information

Biomimetic nanoparticles deliver mRNAs encoding costimulatory receptors and enhance T cell mediated cancer immunotherapy

Wenqing Li[1,10], Xinfu Zhang[1,2,10], Chengxiang Zhang[1,10], Jingyue Yan[1,10], Xucheng Hou[1], Shi Du[1], Chunxi Zeng[1], Weiyu Zhao[1], Binbin Deng[3], David W. McComb[3,4], Yuebao Zhang[1], Diana D. Kang[1], Junan Li[1], William E. Carson III[5], Yizhou Dong[1, 6,7,8,9*]

[1]Division of Pharmaceutics & Pharmacology, College of Pharmacy, The Ohio State University, Columbus, OH 43210, USA.

[2]State Key Laboratory of Fine Chemicals, Dalian University of Technology, Dalian, China.

[3]Center for Electron Microscopy and Analysis, The Ohio State University, Columbus, OH 43212, USA.

[4]Department of Materials Science and Engineering, The Ohio State University, Columbus, OH 43210, USA.

[5]Department of Surgery, Division of Surgical Oncology, The Ohio State University Wexner Medical Center and The OSU James Comprehensive Cancer Center, Columbus, Ohio.

[6]Department of Biomedical Engineering, The Ohio State University, Columbus, OH 43210, USA.

[7]The Center for Clinical and Translational Science, The Ohio State University, Columbus, OH 43210, USA.

[8]The Comprehensive Cancer Center, The Ohio State University, Columbus, OH 43210, USA.

Dorothy M. Davis Heart & Lung Research Institute, The Ohio State University, Columbus, OH 43210, USA.

[9]Department of Radiation Oncology, The Ohio State University, Columbus, OH 43210, USA.

[10]These authors contributed equally: Wenqing Li, Xinfu Zhang, Chengxiang Zhang, Jingyue Yan.

*e-mail: dong.525@osu.edu

Supplementary Methods

Chemical synthesis of phospholipid and glycolipid derivatives (PLs and GLs)

Compounds 3, 5, 7, 8 and their analogues were synthesized according to the methods reported previously[1,2]. General methods for PL1-18 and PL1-GL16 is to a solution of compound i or analogues (0.5 mmole) in CH_2Cl_2 (2 mL) was added excess amount of trifluoroacetic acid (1 mL). The mixture was stirred at RT for 2 h and monitored with thin layer chromatography. Upon completion of the reaction, the solvent was evaporated to yield an oil-like intermediate. The intermediate was dissolved in 10 mL of anhydrous tetrahydrofuran, followed by adding triethylamine (0.2 mL). The resulting mixture was stirred for 30 min at RT. After adding aldehyde (3 mmoles) and $NaBH(OAc)_3$ (3 mmoles), the reaction mixture was stirred at RT for 24 h. After the solvent was removed, the reacting mixture was purified by column chromatography using a CombiFlash Rf system with a RediSep Gold Resolution silica column (Teledyne Isco) with the gradient elution (CH_2Cl_2 and ultra) from 100% CH_2Cl_2 to 70% CH_2Cl_2 (ultra, CH_2Cl_2/MeOH/NH_4OH =75/22/3 by volume) to give the corresponding products.

PL1, yield 34%. ^{1}H NMR (400 MHz, $CDCl_3$) δ = 4.86-4.80 (3H, m), 4.16-4.08 (6H, m), 2.53-2.50 (2H, t, *J* = 8), 2.42-2.37 (8H, m), 2.31-2.28 (6H, t, *J* = 8), 1.83-1.80 (2H, m), 1.63-1.51 (21H, m), 1.37-1.28 (54H, m), 0.90-0.87 (18H, t, *J* = 8). MS (*m/z*): $[M+H]^+$ calcd. for $C_{61}H_{122}N_2O_{10}P$, 1073.88, found, 1073.88.

PL2, yield 64%. ^{1}H NMR (400 MHz, $CDCl_3$) δ = 4.13-4.08 (2H, m), 3.79 (3H, s), 3.76 (3H, s), 2.53-2.50 (2H, t, *J* = 8), 2.42-2.37 (9H, m), 1.85-1.78 (2H, m), 1.62-1.57 (2H, m), 1.45-1.43 (6H, m), 1.27 (54H, s), 0.91-0.87 (9H, t, *J* = 8). MS (*m/z*): $[M+H]^+$ calcd. for $C_{44}H_{94}N_2O_4P$, 745.70, found, 745.69.

PL3, yield 50%. ^{1}H NMR (400 MHz, $CDCl_3$) δ = 4.86-4.80 (3H, m), 4.08-4.03 (2H, m), 2.54-2.51 (2H, t, *J* = 8), 2.46-2.38 (9H, m), 1.83-1.80 (2H, m), 1.62-1.60 (2H, m), 1.45 (8H, m), 1.35-1.34 (12H, m), 1.28 (49H, s), 0.91-0.88 (9H, t, *J* = 8). MS (*m/z*): $[M+H]^+$ calcd. for $C_{48}H_{102}N_2O_4P$, 801.76, found, 801.76.

PL4, yield 48%. ^{1}H NMR (400 MHz, $CDCl_3$) δ = 4.16-4.07 (6H, m), 2.54-2.50 (2H, t, *J* = 8), 2.43-2.37 (9H, m), 1.84-1.80 (2H, m), 1.59-1.56 (2H, m), 1.45 (6H, m), 1.38-1.28 (49H, m), 0.91-0.88 (9H, t, *J* = 8). MS (*m/z*): $[M+H]^+$ calcd. for $C_{40}H_{86}N_2O_4P$, 689.63, found, 689.63.

PL5, yield 40%. ^{1}H NMR (400 MHz, $CDCl_3$) δ = 4.14-4.07 (6H, m), 2.54-2.50 (2H, t, *J* = 8), 2.43-2.37 (8H, m), 1.84-1.80 (2H, m), 1.59-1.56 (2H, m), 1.45 (6H, m), 1.37-1.28 (55H, m), 0.91-0.88 (9H, t, *J* = 8). MS (*m/z*): $[M+H]^+$ calcd. for $C_{43}H_{92}N_2O_4P$, 731.68, found, 731.68.

PL6, yield 48%. ^{1}H NMR (400 MHz, $CDCl_3$) δ = 4.16-4.07 (6H, m), 3.72-3.69 (2H, m), 2.54-2.50 (2H, t, *J* = 8), 2.44-2.38 (8H, m), 1.86-1.79 (2H, m), 1.72-1.69 (2H, m), 1.44 (6H, m), 1.37-1.34 (6H, m), 1.27 (54H, s), 0.91-0.88 (9H, t, *J* = 8). MS (*m/z*): $[M+H]^+$ calcd. for $C_{46}H_{98}N_2O_4P$, 773.73, found, 773.73.

PL7, yield 41%. ^{1}H NMR (400 MHz, $CDCl_3$) δ = 4.16-4.07 (6H, m), 3.72-3.69 (2H, m), 2.54-2.50 (2H, t, *J* = 8), 2.44-2.38 (8H, m), 1.86-1.79 (2H, m), 1.72-1.69 (2H, m), 1.44 (6H, m), 1.37-1.34 (6H, m), 1.27 (54H, s), 0.91-0.88 (9H, t, *J* = 8). MS (*m/z*): $[M+H]^+$ calcd. for $C_{49}H_{104}N_2O_4P$, 815.77, found, 815.77.

PL8, yield 26%. ^{1}H NMR (400 MHz, $CDCl_3$) δ = 4.14-4.08 (6H, m), 3.24-3.22 (2H, m), 2.80-2.77 (1H, t, *J* = 8), 2.54-2.50 (2H, t, *J* = 8), 2.46-2.32 (14H, m), 2.22 (3H, s), 1.83-1.80 (2H, m), 1.65-1.60 (6H, m), 1.44 (5H, m), 1.37-1.28 (50H, m), 0.91-0.88 (9H, t, *J* = 8). MS (*m/z*): $[M+H]^+$ calcd. for $C_{44}H_{95}N_3O_4P$, 760.71, found, 760.71.

PL9, yield 24%. ^{1}H NMR (400 MHz, $CDCl_3$) δ = 4.16-4.07 (6H, m), 2.80-2.76 (2H, t, *J* = 8), 2.74-2.70 (4H, m), 2.61-2.58 (2H, m), 2.53-2.44 (8H, m), 2.31 (3H, s), 1.87-1.81 (4H, m), 1.69-1.66 (2H, m), 1.56 (4H, m), 1.44 (2H, m), 1.37-1.27 (54H, m), 0.91-0.87 (9H, t, *J* = 8). MS (*m/z*): $[M+H]^+$ calcd. for $C_{47}H_{101}N_3O_4P$, 802.75, found, 802.75.

PL10, yield 41%. ^{1}H NMR (400 MHz, $CDCl_3$) δ = 4.12-4.06 (6H, m), 2.51-2.50 (2H, t, *J* = 4), 2.43-2.32 (14H, m), 2.22 (3H, s), 1.83-1.79 (2H, m), 1.62-1.60 (2H, m), 1.43 (6H, m), 1.37-1.27 (62H, m), 0.91-0.87 (9H, t, *J* = 8). MS (*m/z*): $[M+H]^+$ calcd. for $C_{50}H_{107}N_3O_4P$, 844.80, found, 844.80.

PL11, yield 33%. ^{1}H NMR (400 MHz, $CDCl_3$) δ = 4.15-4.06 (6H, m), 2.53-2.50 (2H, t, *J* = 4), 2.44-2.40 (9H, m), 2.37-2.32 (5H, m), 2.22 (3H, s), 1.83-1.79 (2H, m), 1.71-1.68 (1H, m), 1.64-1.60 (4H, m), 1.43 (6H, m), 1.37-1.27 (66H, m), 0.91-0.87 (9H, t, *J* = 8). MS (*m/z*): $[M+H]^+$ calcd. for $C_{53}H_{113}N_3O_4P$, 886.85, found, 886.85.

PL12, yield 32%. ^{1}H NMR (400 MHz, $CDCl_3$) δ = 4.15-4.06 (6H, m), 2.52-2.31 (22H, m), 1.84-1.77 (2H, m), 1.65-1.60 (4H, m), 1.42-1.41 (6H, m), 1.37-1.27 (49H, m), 0.91-0.87 (9H, t, *J* = 8). MS (*m/z*): $[M+H]^+$ calcd. for $C_{47}H_{100}N_4O_4P$, 815.75, found, 815.75.

PL13, yield 30%. ^{1}H NMR (400 MHz, $CDCl_3$) δ = 4.16-4.06 (6H, m), 2.52-2.32 (22H, m), 1.82-1.79 (2H, m), 1.66-1.60 (4H, m), 1.42-1.41 (6H, m), 1.37-1.27 (55H, m), 0.91-0.87 (9H, t, *J* = 8). MS (*m/z*): $[M+H]^+$ calcd. for $C_{50}H_{106}N_4O_4P$, 857.80, found, 857.79.

PL14, yield 36%. ^{1}H NMR (400 MHz, $CDCl_3$) δ = 4.16-4.07 (6H, m), 2.53-2.32 (22H, m), 1.83-1.80 (2H, m), 1.66-1.61 (4H, m), 1.42 (6H, m), 1.37-1.28 (61H, m), 0.91-0.88 (9H, t, *J* = 8). MS (*m/z*): $[M+H]^+$ calcd. for $C_{53}H_{112}N_4O_4P$, 899.84, found, 899.84.

PL15, yield 21%. ^{1}H NMR (400 MHz, $CDCl_3$) δ = 4.13-4.08 (6H, m), 2.53-2.33 (24H, m), 1.85-1.80 (4H, m), 1.66-1.63 (5H, m), 1.42 (9H, m), 1.38-1.28 (72H, m), 0.91-0.88 (9H, t, *J* = 8). MS (*m/z*): $[M+H]^+$ calcd. for $C_{56}H_{118}N_4O_4P$, 941.89, found, 941.89.

PL16, yield 23%. ^{1}H NMR (400 MHz, $CDCl_3$) δ = 5.69-5.63 (3H, m), 5.57-5.51 (3H, m), 4.65-4.63 (6H, d, *J* = 8), 4.16-4.08 (6H, m), 2.55-2.40 (10H, m), 2.34-2.30 (6H, m), 2.14-2.09 (6H, m), 1.85-1.80 (2H, m), 1.65-1.62 (9H, m), 1.42 (9H, m), 1.38-1.31 (62H, m), 0.92-0.89 (9H, t, *J* = 8). MS (*m/z*): $[M+H]^+$ calcd. for $C_{64}H_{122}N_2O_{10}P$, 1109.87, found, 1109.89.

PL17, yield 23%. ^{1}H NMR (400 MHz, $CDCl_3$) δ = 5.41-5.28 (12H, m), 4.15-4.06 (6H, d, *J* = 8), 3.15-3.03 (2H, m), 2.97-2.89 (7H, m), 2.78-2.75 (7H, m), 2.07-2.00 (22H, m), 1.62-1.55 (5H, m), 1.35-1.29 (51H, m), 0.90-0.86 (9H, t, *J* = 8). MS (*m/z*): $[M+H]^+$ calcd. for $C_{64}H_{122}N_2O_4P$, 1013.91, found, 1013.91.

PL18, yield 24%. ^{1}H NMR (400 MHz, $CDCl_3$) δ = 4.22-4.10 (7H, m), 2.43-2.39 (11H, m), 2.34-2.30 (2H, t, *J* = 8), 2.04-2.01 (2H, t, *J* = 8), 1.67-1.63 (4H, m), 1.38-1.28 (71H, m), 0.91-0.88 (9H, t, *J* = 8). MS (*m/z*): $[M+H]^+$ calcd. for $C_{52}H_{107}N_2O_6P$, 887.79, found, 887.79.

GL1, yield 26%. ^{1}H NMR (400 MHz, $CDCl_3$) δ = 4.17-4.11 (2H, m), 2.70-2.57 (11H, m), 2.33-2.29 (2H, t, *J* = 8), 1.78 (2H, s), 1.69-1.62 (3H, m), 1.54-1.48 (8H, m), 1.28 (59H, s), 0.92-0.88 (9H, t, *J* = 8). MS (*m/z*): $[M+H]^+$ calcd. for $C_{50}H_{95}N_2O_{10}$, 883.70; found, 883.70.

GL2, yield 35%. ^{1}H NMR (400 MHz, $CDCl_3$) δ = 5.40 (1H, m), 5.24-5.19 (1H, m), 5.04-5.00 (1H, m), 4.48-4.46 (1H, d, *J* = 8), 4.17 (2H, m), 3.91 (2H, m), 3.54-3.51 (1H, m), 2.40-2.38 (12H, m), 2.16 (3H, s), 2.06 (6H, s), 1.99 (4H, s), 1.74 (2H, m), 1.73-1.70 (2H, m), 1.57 (6H, m), 1.27 (52H, s), 0.89 (9H, t, *J* = 8). MS (*m/z*): $[M+H]^+$ calcd. for $C_{53}H_{101}N_2O_{10}$, 925.75, found, 925.74.

GL3, yield 64%. ^{1}H NMR (400 MHz, $CDCl_3$) δ = 5.41-5.40 (1H, m), 5.32-5.20 (1H, m), 5.04-5.01 (1H, m), 4.48-4.46 (1H, d, *J* = 8), 4.22-4.13 (2H, m), 3.94-3.90 (2H, m), 3.54-3.52 (1H, m), 2.46-2.37 (12H, m), 2.16 (3H, s), 2.06 (6H, s), 2.00 (4H, s), 1.73-1.72 (2H, m), 1.58-1.56 (2H, m), 1.42 (6H, m), 1.28 (55 H, s), 0.89 (9H, t, *J* = 8). MS (*m/z*): $[M+H]^+$ calcd. for $C_{56}H_{107}N_2O_{10}$, 967.79, found, 967.79.

GL4, yield 35%. ^{1}H NMR (400 MHz, $CDCl_3$) δ = 5.39 (1H, m), 5.19-5.15 (1H, m), 5.03-5.01 (1H, m), 4.47-4.45 (1H, m), 4.15-4.14 (2H, m), 3.93-3.92 (2H, m), 3.53-3.51 (1H, m), 2.84-2.74 (6H, m), 2.64-2.59 (4H, m), 2.55-2.51 (2H, m), 2.10 (3H, s), 2.05 (6H, s), 1.98 (6H, s), 1.83-1.78 (4H, m), 1.58 (4H, m), 1.46 (2H, m), 1.26 (63 H, s), 0.89-0.88 (9H, t, *J* = 4). MS (*m/z*): $[M+H]^+$ calcd. for $C_{59}H_{113}N_2O_{10}$, 1009.84, found, 1009.84.

GL5, yield 35%. ^{1}H NMR (400 MHz, $CDCl_3$) δ = 5.41-5.40 (1H, m), 5.22-5.19 (1H, m), 5.04 (1H, m), 4.48-4.46 (1H, d, *J* = 8), 4.17 (2H, m), 3.91 (2H, m), 3.54-3.52 (1H, m), 2.84-2.51 (15H, m), 2.10 (3H, s), 2.16 (3H, s), 2.07 (5H, s), 2.00 (4H, s), 1.73-1.72 (2H, m), 1.62-1.60 (4H, s), 1.43-1.42 (6H, m), 1.26 (53H, s), 0.89-0.88 (9H, t, *J* = 8). MS (*m/z*): $[M+H]^+$ calcd. for $C_{60}H_{116}N_3O_{10}$, 1038.87, found, 1038.86.

GL6, yield 35%. ^{1}H NMR (400 MHz, $CDCl_3$) δ = 5.41-5.40 (1H, m), 5.24-5.21 (1H, m), 5.04=5.01 (1H, m), 4.48-4.46 (1H, d, J = 8), 4.22-4.12 (2H, m), 3.95-3.89 (2H, m), 3.56-3.50 (1H, m), 2.84-2.33 (22H, m), 2.16 (3H, s), 2.07-2.06 (1H, s), 2.00 (3H, s), 1.73-1.63 (6H, m), 1.42 (6H, m), 1.27 (53H, s), 0.91-0.88 (9H, t, J = 8). MS (*m/z*): $[M+H]^+$ calcd. for $C_{63}H_{121}N_4O_{10}$, 1093.91, found, 1093.91.

GL7, yield 30%. ^{1}H NMR (400 MHz, $CDCl_3$) δ = 5.24-5.19 (1H, m), 5.13-5.08 (1H, m), 5.02-4.98 (1H, m), 4.52-4.50 (1H, d, J = 8), 4.32-4.27 (1H, m), 4.16-4.13 (1H, m), 3.94-3.89 (1H, m), 3.72-3.69 (1H, m), 3.56-3.50 (1H, m), 3.37-3.34 (1H, m), 2.46-2.35 (15H, m), 2.23 (3H, s), 2.10-2.02 (11H, m), 1.72-1.60 (8H, m), 1.45-1.28 (64H, s), 0.91-0.88 (12H, t, J = 8). MS (*m/z*): $[M+H]^+$ calcd. for $C_{60}H_{116}N_3O_{10}$, 1038,87, found, 1038.87.

GL8, yield 65%. ^{1}H NMR (400 MHz, $CDCl_3$) δ = 5.24-5.19 (1H, m), 5.12-5.08 (1H, m), 5.01-4.97 (1H, m), 4.51-4.49 (1H, d, J = 8), 4.31-4.27 (1H, m), 4.16-4.13 (1H, m), 3.92-3.88 (1H, m), 3.70-3.69 (3H, m), 3.55-3.50 (1H, m), 2.47-2.34 (25H, m), 2.10 (3H, s), 2.05-2.02 (9H, m), 1.70 (10H, m), 1.50 (10H, m), 1.27 (55H, s), 0.91-0.88 (9H, t, J = 8). MS (*m/z*): $[M+H]^+$ calcd. for $C_{63}H_{121}N_4O_{10}$, 1093,91, found, 1093. 91.

GL9, yield 49%. ^{1}H NMR (400 MHz, $CDCl_3$) δ = 5.69-5.63 (1H, m), 5.57-5.51 (3H, m), 5.24-5.19 (1H, m), 5.12-5.08 (1H, m), 5.02-4.97 (1H, m), 4.64-4.63 (6H, d, J = 4), 4.52-4.50 (1H, d, J = 8), 4.31-4.27 (1H, m), 4.17-4.11 (1H, m), 3.94-3.88 (1H, m), 3.73-3.69 (1H, m), 3.54-3.51 (1H, m), 2.47-2.30 (18H, m), 2.14-2.02 (17H, m), 1.65-1.62 (11H, m), 1.40-1.31 (55H, m), 0.92-0.88 (9H, t, J = 8). MS (*m/z*): $[M+H]^+$ calcd. for $C_{74}H_{131}N_2O_{16}$, 1303.95, found, 1303.94.

GL10, yield 33%. ^{1}H NMR (400 MHz, $CDCl_3$) δ = 5.64-5.63 (2H, m), 5.57-5.51 (2H, m), 5.24-5.19 (1H, m), 5.13-5.08 (1H, m), 5.02-4.97 (1H, m), 4.64-4.63 (4H, d, J = 4), 4.52-4.50 (1H, d, J = 8), 4.31-4.27 (1H, m), 4.17-4.13 (1H, m), 3.95-3.89 (1H, m), 3.72-3.70 (2H, m), 3.58-3.52 (2H, m), 2.55-2.38 (9H, m), 2.34-2.30 (5H, m), 2.22 (2H, m), 2.16-2.02 (16H, m), 1.79-1.60 (9H, m), 1.46-1.27 (32H, m), 0.92-0.88 (6H, t, J = 8). MS (*m/z*): $[M+H]^+$ calcd. for $C_{57}H_{101}N_2O_{14}$, 1037.73, found, 1037.73.

GL11, yield 20%. ^{1}H NMR (400 MHz, $CDCl_3$) δ = 5.25-5.20 (1H, m), 5.14-5.08 (1H, m), 5.02-4.97 (1H, m), 4.55-4.53 (1H, d, *J* = 8), 4.32-4.28 (1H, m), 4.17-4.13 (1H, m), 4.08-4.05 (6H, t, *J* = 8), 3.93-3.88 (1H, m), 3.75-3.71 (1H, s), 3.57-3.53 (1H, m), 2.80-2.76 (6H, t, *J* = 8), 2.47-2.43 (10H, m), 2.11 (3H, s), 2.07 (3H, s), 2.04 (3H, s), 2.02 (3H, s), 1.67-1.60 (12H, m), 1.32-1.28 (54H, m), 0.92-0.88 (9H, t, *J* = 8). MS (*m/z*): $[M+H]^+$ calcd. for $C_{65}H_{119}N_2O_{16}$, 1183.86, found, 1183.85.

GL12, yield 63%. ^{1}H NMR (400 MHz, $CDCl_3$) δ = 5.24-5.19 (1H, m), 5.12-5.08 (1H, m), 5.02-4.97 (1H, m), 4.52-4.50 (1H, d, *J* = 8), 4.31-4.26 (1H, m), 4.16-4.12 (1H, m), 3.94-3.89 (1H, m), 3.71-3.68 (2H, t, *J* = 8), 3.58-3.52 (1H, m), 2.44-2.33 (12H, t, *J* = 8), 2.21 (3H, s), 2.10 (3H, s), 2.06 (3H, s), 2.04 (3H, s), 2.02 (3H, s), 1.75-1.63 (6H, m), 1.44 (4H, m), 1.27 (37H, m), 0.91-0.87 (6H, t, *J* = 8). MS (*m/z*): $[M+H]^+$ calcd. for $C_{45}H_{85}N_2O_{10}$, 813.62, found, 813.62.

GL13, yield 44%. ^{1}H NMR (400 MHz, $CDCl_3$) δ = 5.42 (1H, m), 5.24-5.19 (1H, m), 5.13-5.09 (1H, m), 4.52-4.50 (1H, m), 4.21-4.17 (1H, m), 4.07-3.87 (3H, m), 3.53-3.47 (1H, m), 2.52-2.39 (10H, m), 1.77-1.66 (4H, m), 1.50-1.42 (5H, m), 1.27-1.13 (89H, m), 0.91-0.87 (9H, t, *J* = 8). MS (*m/z*): $[M+H]^+$ calcd. for $C_{68}H_{131}N_2O_{10}$, 1135.98, found, 1135.98.

GL14, yield 37%. ^{1}H NMR (400 MHz, $CDCl_3$) δ = 5.43-5.24 (4H, m), 5.09-5.04 (1H, t, *J* = 8), 4.89-4.82 (2H, m), 4.53-4.47 (2H, m), 4.29-4.22 (3H, m), 4.07-3.96 (4H, m), 3.90-3.87 (1H, m), 3.72-3.67 (4H, m), 3.53-3.50 (1H, m), 2.48-2.37 (10H, m), 2.16-2.01 (27H, m), 1.73-1.69 (3H, m), 1.45-1.41 (6H, m), 1.27 (45H, s), 0.91-0.87 (9H, t, *J* = 8). MS (*m/z*): $[M+H]^+$ calcd. for $C_{68}H_{123}N_2O_{18}$, 1255.88, found, 1255.88.

GL15, yield 48%. ^{1}H NMR (400 MHz, $CDCl_3$) δ = 5.36-5.35 (1H, d, *J* = 4), 5.22-5.18 (1H, t, *J* = 8), 5.14-5.10 (1H, m), 4.98-4.95 (1H, m), 4.91-4.87 (1H, m), 4.50-4.45 (3H, m), 4.15-4.07 (3H, m), 3.88-3.78 (4H, m), 3.62-3.58 (1H, m), 3.52-3.47 (1H, m), 2.39-2.37 (1H, m), 2.16 (3H, s), 2.13 (3H, s), 2.07-2.04 (12H, m), 1.97 (3H, s), 1.71-1.68 (3H, m), 1.55-1.53 (2H, m), 1.41 (6H, m), 1.27 (51H, m), 0.91-0.87 (9H, t, *J* = 8). MS (*m/z*): $[M+H]^+$ calcd. for $C_{68}H_{123}N_2O_{18}$, 1255.88, found, 1255.88.

GL16, yield 55%. ^{1}H NMR (400 MHz, $CDCl_3$) δ = 5.34-5.30 (1H, m), 5.24-5.23 (1H, m), 4.99 (1H, m), 4.35-4.28 (2H, m), 4.14-4.10 (1H, m), 3.75-3.71 (1H, t, *J* = 8), 3.45-3.41 (1H, t, *J* = 8), 2.41(12H, m), 2.12-2.05 (9H, m), 1.70(3H, m), 1.58 (3H, m), 1.42 (6H, m), 1.27 (52H, s), 0.91-0.87 (9H, t, *J* = 8). MS (*m/z*): $[M+H]^+$ calcd. for $C_{53}H_{103}N_2O_8$, 895.77, found, 895.77.

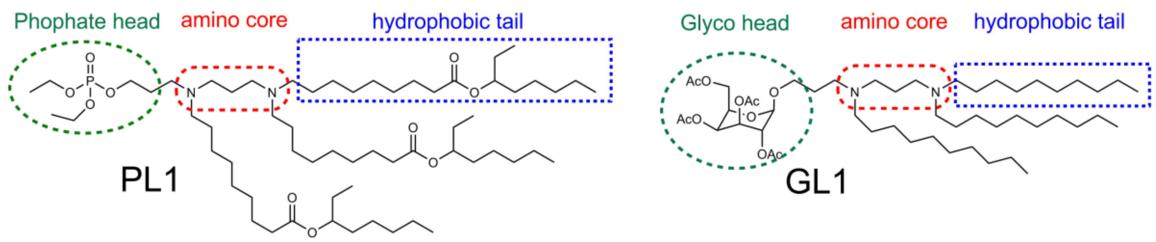

Supplementary Figure 1. Structures of biomimetic lipids: phospholipid (PL) and glycolipid (PL) derivatives. The representative examples PL1 and GL1 are composed of a biomimetic head (phosphate head or glyco head), an ionizable amino core, and multiple hydrophobic tails.

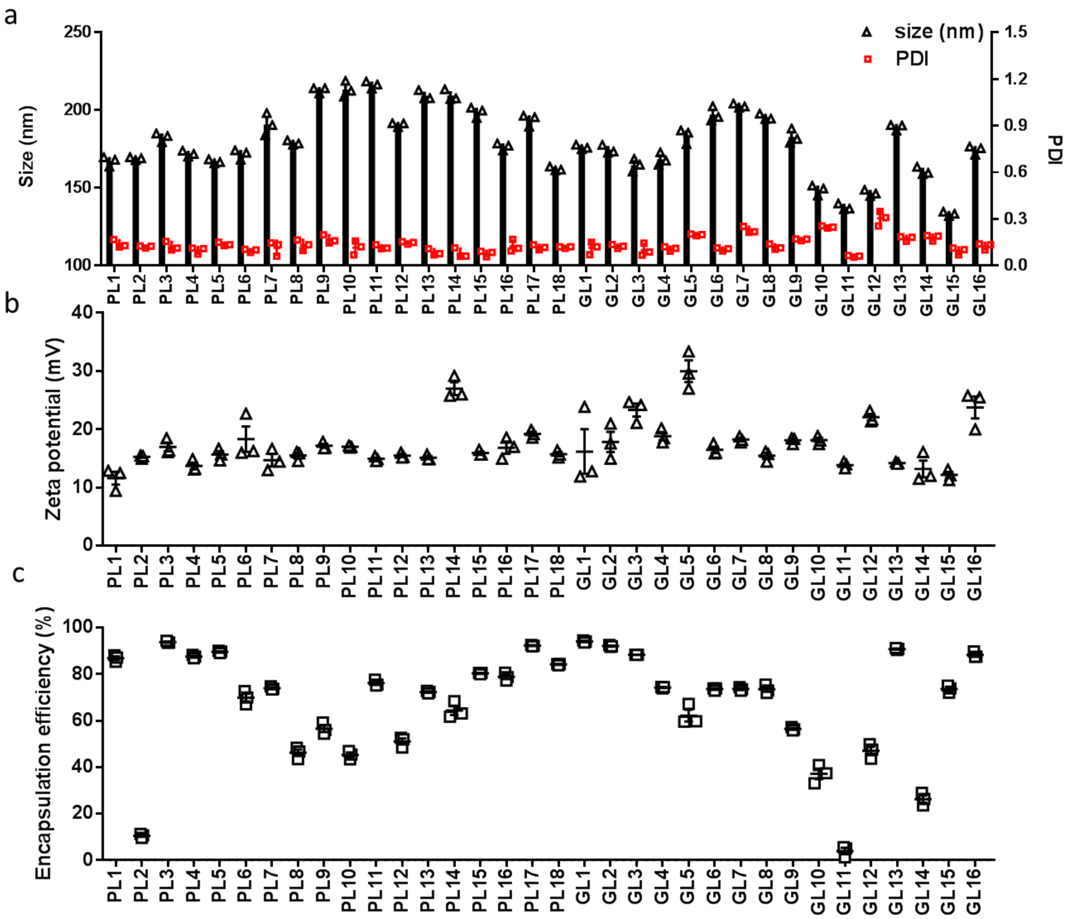

Supplementary Figure 2. Characterizations of phospholipid (PL) and glycolipid (GL) derived nanoparticles. a, Nanoparticle size (nm) and polydispersity index (PDI). **b**, Zeta potential (mV). **c**, Entrapment efficiency of Fluc mRNA. All data are from n = 3 biologically independent samples and present as the mean ± S.E.M. Source data are provided as a Source Data file.

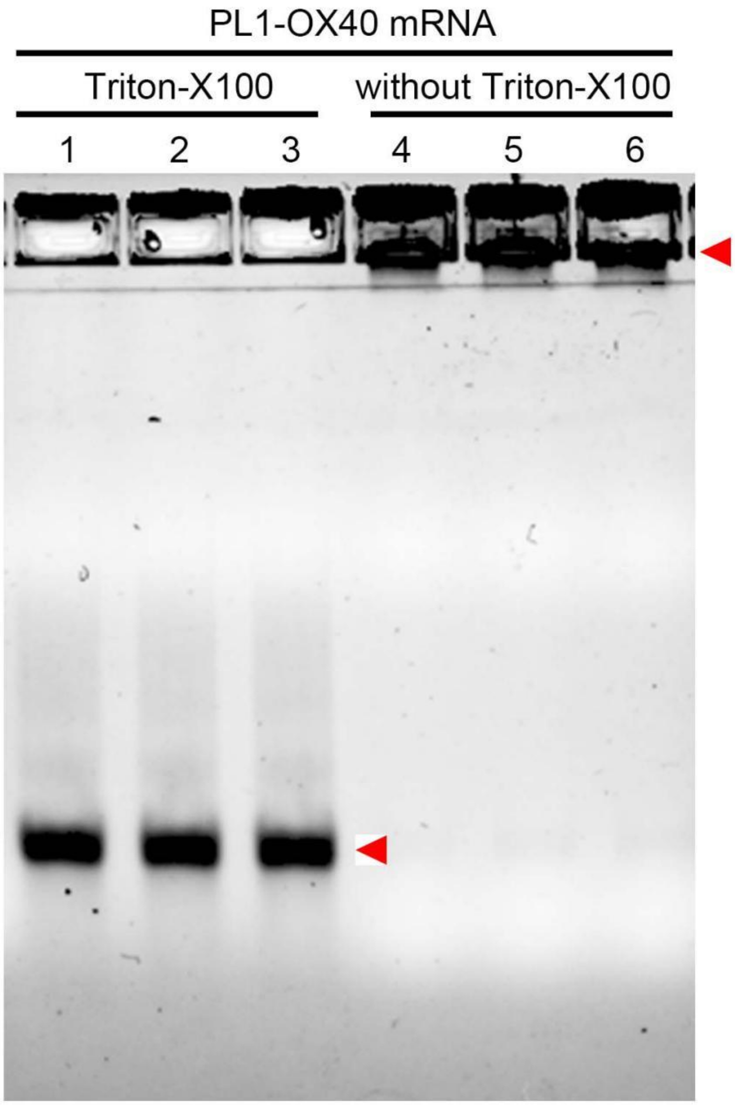

Supplementary Figure 3. The gel electrophoresis of PL1-OX40 mRNA treated with/without triton. Line 1-3 are PL1-OX40 mRNA nanoparticles treated with Triton-X100. Line 4-6 are PL1-OX40 mRNA nanoparticles without Triton-X100. Data presented are from a single experiment.

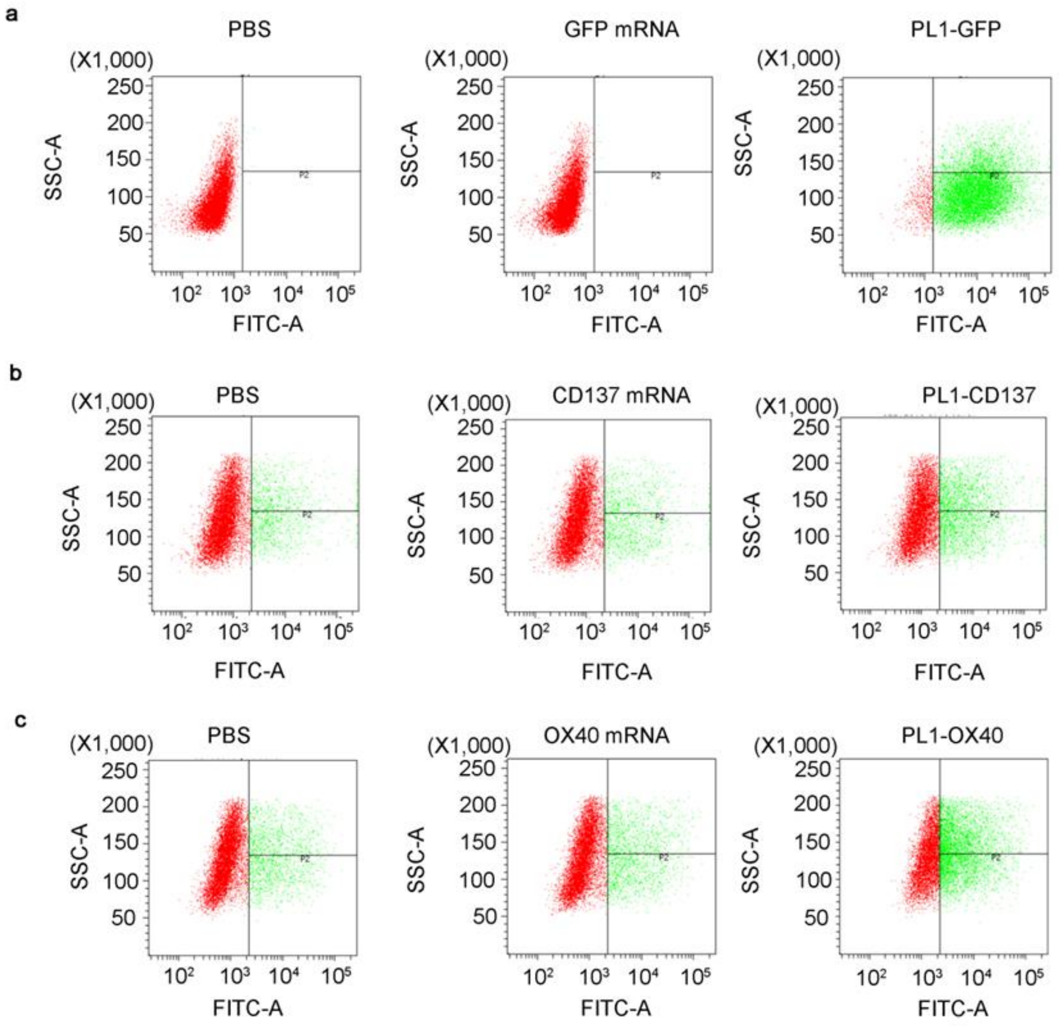

Supplementary Figure 4. Flow cytometry analysis of PL1 mediated mRNA delivery in E.G7 cells. a, PL1 nanoparticles delivered GFP mRNA to E.G7 cells, related to of Fig. 2c. **b**, PL1-CD137 induced CD137 expression in E.G7 cells, related to Fig. 2d. **c**, PL1-OX40 induced OX40 expression in EG.7 cells, related to Fig. 2e. Gate P2 are GFP positive or OX40/CD137 positive cells.

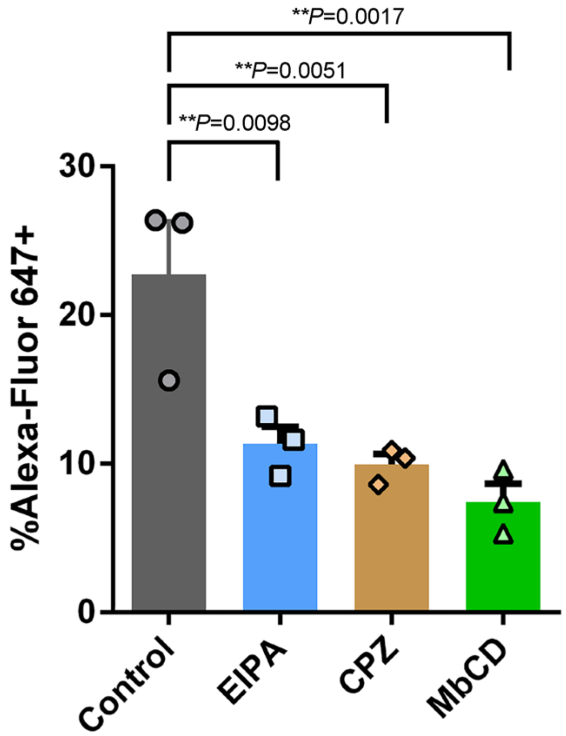

Supplementary Figure 5. Endocytic pathways of the PL1 nanoparticles. E.G7 cells were treated with 5-(N-Methyl-N-isopropyl)amiloride (EIPA), chlorpromazine hydrochloride (CPZ), or methyl-β-cyclodextrin (MβCD). After 0.5 h, cells were treated with PL1-Alexa-Fluor 647-labeled RNA nanoparticles. After 3 h, cells were analyzed by flow cytometry. All data are from n = 3 biologically independent samples and are presented as the mean ± S.E.M. Statistical significance was analyzed using one-way ANOVA followed by Dunnett's multiple comparisons test. $^{**}P < 0.01$. Source data are provided as a Source Data file.

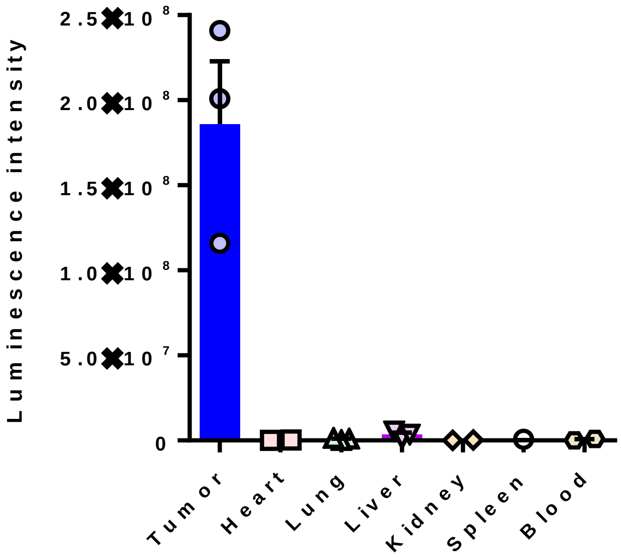

Supplementary Figure 6. Quantification of luminescence intensity after i.t. injection of PL1-Fluc luciferase mRNA. C57BL/6 mice were s.c. implanted with B16F10 melanoma cells. The tumor was i.t. injected with PL1-Fluc (20 μg mRNA/mouse). After 24 h, the tissues were collected and imaged by a Xenogen IVIS spectrum imaging system (n = 3). Data are presented as the mean ± S.E.M. Source data are provided as a Source Data file.

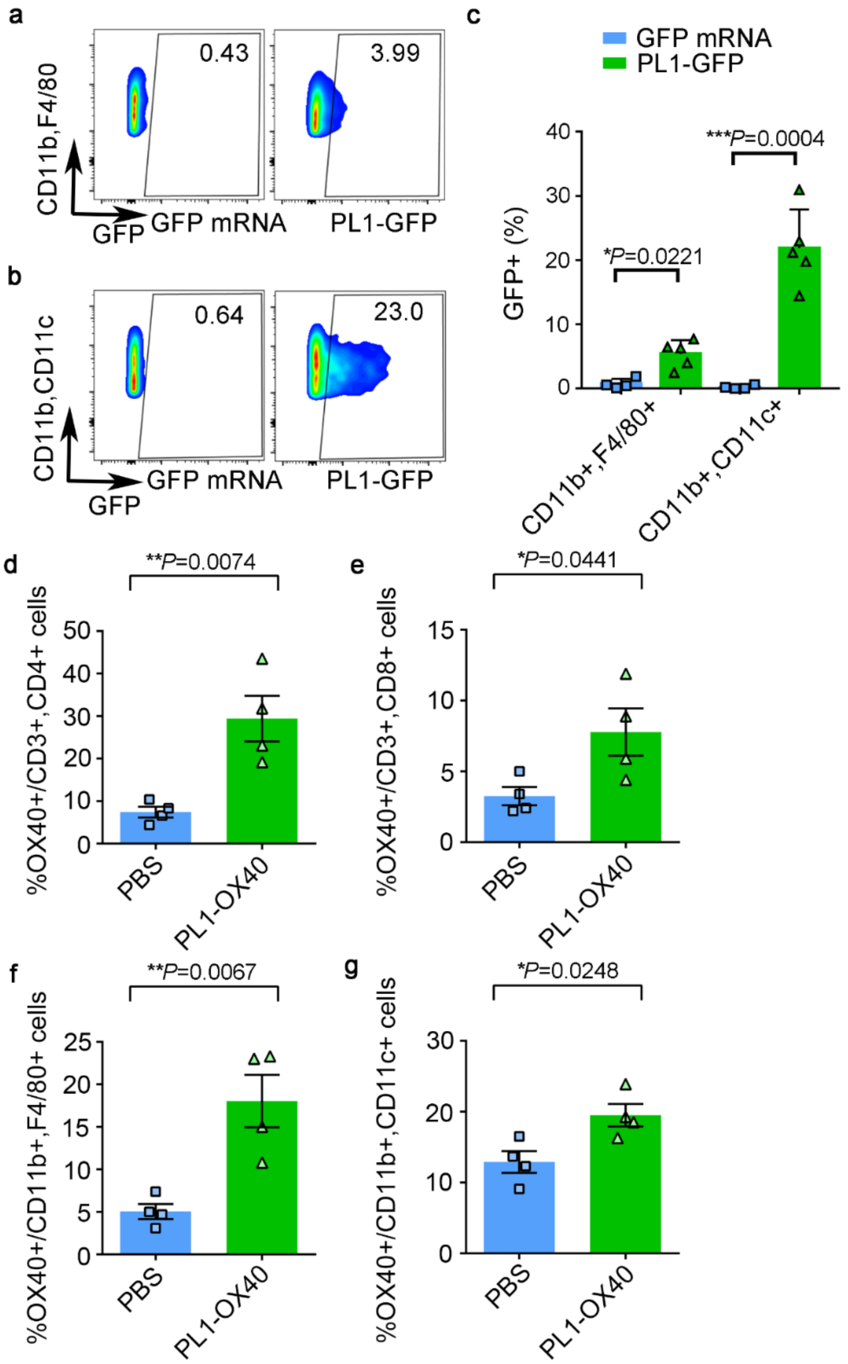

Supplementary Figure 7. GFP and OX40 expression in B16F10 tumors after a single injection of PL1-GFP mRNA or PL1-OX40 mRNA. a, b, c, GFP expression in Macrophages and dendritic cells after a single i.t. injection with GFP mRNA (n=4) or PL1-GFP mRNA (n=5) in tumor microenvironment. **d**, **e**, **f**, **g**, The OX40 expression in CD4+, CD8+ T cells, Macrophages and

dendritic cells after a single intratumoral injection with PBS (n=4) or PL1-OX40 mRNA (n=4) in B16F10 tumors. Data in **c**, **d**, **e**, **f** and **g** are presented as the mean ± S.E.M. Statistical significance in **c, d, e, f and g** were analyzed with two-tailed Student's *t* test. *$P < 0.05$; **$P < 0.01$; ***$P < 0.001$. Source data are provided as a Source Data file.

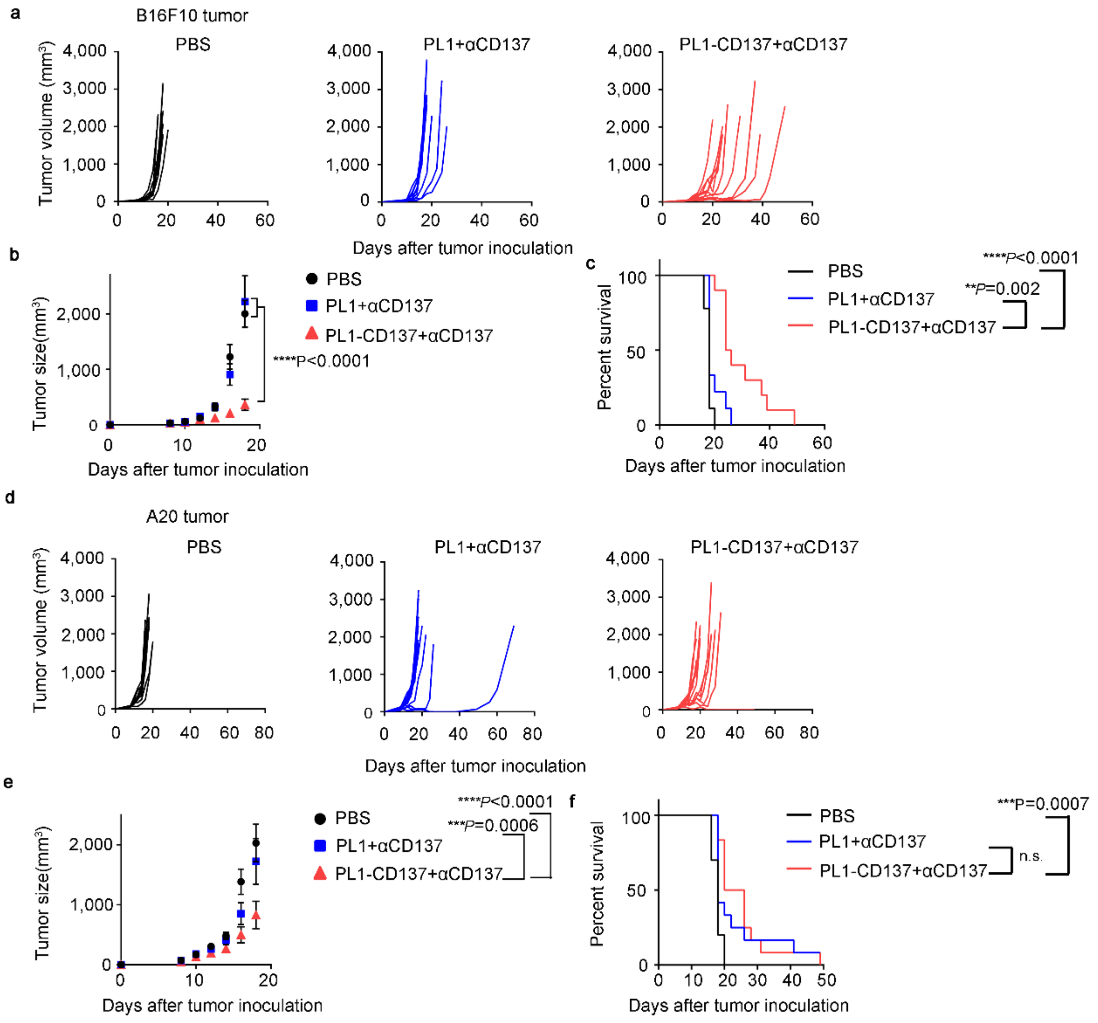

Supplementary Figure 8. Regression of mouse tumors after treatment with PL1-CD137 mRNA + anti-CD137 Ab. a, C57BL/6 mice were implanted subcutaneously with B16F10 melanoma cells. Tumor volumes of individual mice treated with PBS (n=10), PL1+anti-CD137 Ab (n=10), or PL1-CD137+anti-CD137 Ab (n=10) treatment. PL1-CD137 (10 μg mRNA/mouse) were i.t. injected. After 4 h, anti-CD137 Ab (16 μg/mouse) were i.t. injected. Six i.t. doses were given every other day. **b**, **c**, C57BL/6 mice were implanted s.c. with B16F10 melanoma cells. Tumor size (**b**) and survival (**c**) of mice (n=10 per group) after PBS, PL1+anti-CD137 Ab or PL1-CD137+anti-CD137 Ab treatments. **d**, BALB/c mice were implanted subcutaneously with A20 lymphoma cells. Tumor volumes of individual mice treated with PBS (n=10), PL1+anti-CD137 Ab (n=12), and PL1-

CD137+anti-CD137 Ab (n=12). PL1-CD137 (10 μg mRNA/mouse) were i.t. injected, after 4 h, anti-CD137 Ab (16 μg/mouse) were i.t. injected. Six i.t. doses were given every other day. **e**, **f**, BALB/c mice were implanted s.c. with A20 lymphoma cells. Tumor volume (**e**) and survival (**f**) of mice treated with PBS (n=10), PL1+anti-CD137 Ab (n=12) or PL1-CD137+anti-CD137 Ab (n=12). Data in **b** and **e** are presented as the mean ± S.E.M. Statistical significance in **b** and **e** were analyzed by the two-way ANOVA. Statistical significance in **c** and **f** were analyzed using the log-rank (Mantel-Cox) test. $^{**}P < 0.01$; $^{***}P < 0.001$; $^{****}P < 0.0001$; n.s., not significant. Source data are provided as a Source Data file.

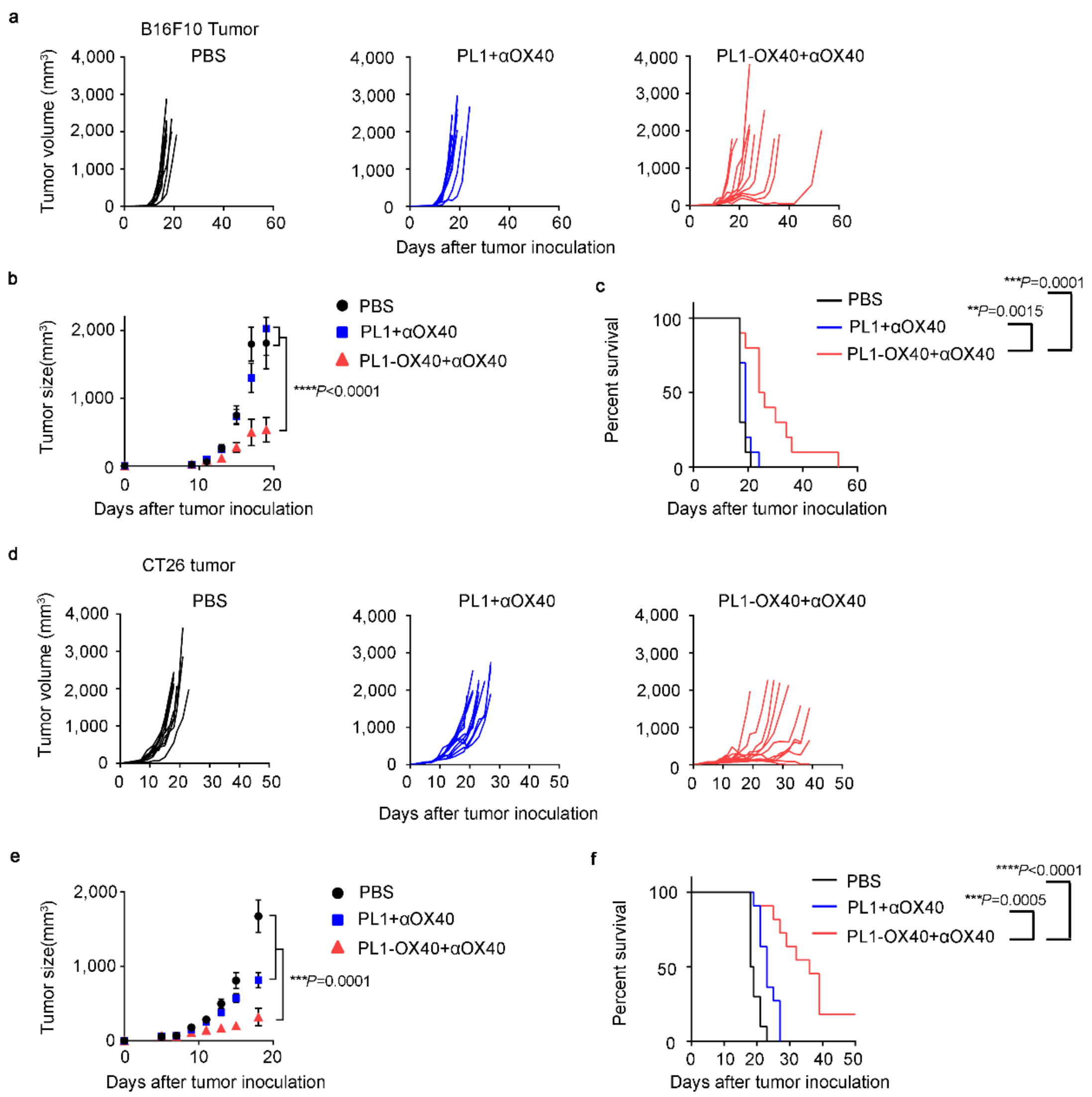

Supplementary Figure 9. Regression of mouse tumors after treatment with PL1-OX40 mRNA + anti-OX40 Ab. a, C57BL/6 mice were implanted subcutaneously with B16F10 melanoma cells. Tumor volumes of individual animals treated with PBS (n=10), PL1+anti-OX40 Ab (n=10) or PL1-OX40+anti-OX40 Ab (n=10). PL1-OX40 (10 µg mRNA/mouse) were i.t. injected, after 4 h, anti-OX40 Ab (8 µg/mouse) were i.t. injected. Six i.t. doses were given every other day. **b**, **c**, C57BL/6 mice bearing B16F10 melanoma cells. Tumor size (**b**) and survival (**c**) of mice (n=10 per group) treated with PBS, PL1+anti-OX40 Ab or PL1-OX40+anti-OX40 Ab treatments. **d**, BABL/c mice were implanted subcutaneously with CT26 cells. Tumor volumes of individual animals treated with

PBS (n=10), PL1+anti-OX40 Ab (n=11), and PL1-OX40+anti-OX40 Ab (n=11). PL1-OX40 (10 μg mRNA/mouse) were i.t. injected, after 4 h, anti-OX40 Ab (8 μg/mouse) were i.t. injected. Six i.t. doses were given every other day. **e**, **f**, BABL/c mice were implanted subcutaneously with CT26 colon carcinoma cells. Tumor size (**e**) and survival (**f**) of mice (n= 10-11 per group) treated with PBS, PL1+anti-OX40 Ab or PL1-OX40+anti-OX40 Ab. Data in **b** and **e** are presented as the mean ± S.E.M. Statistical significance in **b** and **e** were analyzed by the two-way ANOVA. Statistical significance in **c** and **f** were analyzed using the log-rank (Mantel-Cox) test. **$P < 0.01$; ***$P < 0.001$; ****$P < 0.0001$. Source data are provided as a Source Data file.

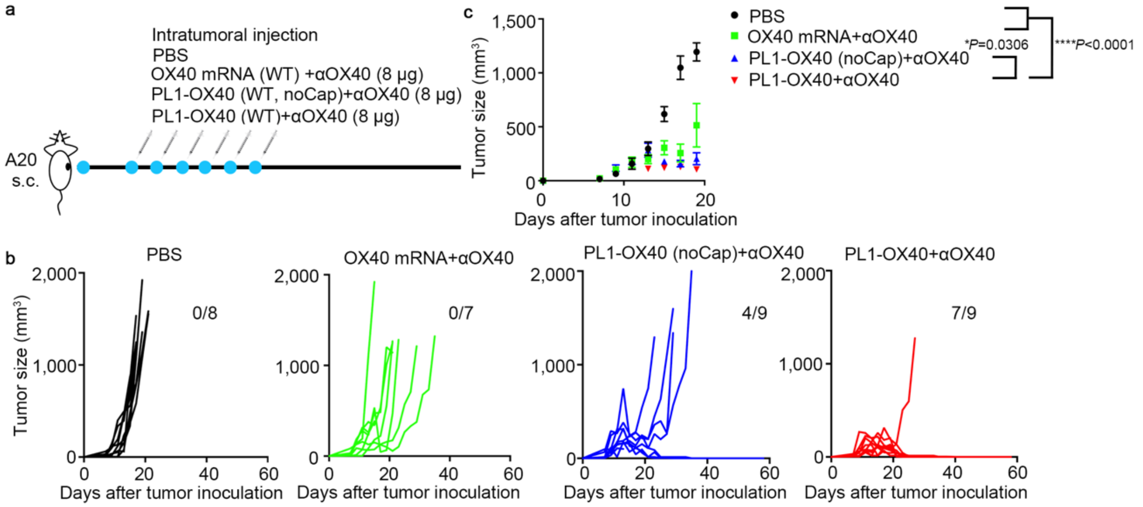

Supplementary Figure 10. Regression of A20 tumors. a, Schematic illustration of the A20 mouse tumor model and the treatment regimen. **b**, Tumor volumes of individual mice (n=7-9 per group) after i.t. injected six doses of PBS (n=8), OX40 mRNA +anti-OX40 Ab (n=7), PL1-OX40 (nocap)+anti-OX40 Ab (n=9), or PL1-OX40+anti-OX40 Ab (n=9). PL1-OX40 (nocap) (10 µg mRNA/mouse), PL1-OX40 (10 µg mRNA/mouse), and anti-OX40 Ab (8 µg/mouse). **c**, Tumor volumes. Data in **c** is presented as the mean ± S.E.M. n=8 mice in PBS group, n=7 mice in OX40 mRNA +anti-OX40 Ab group, n=9 mice in PL1-OX40 (nocap)+anti-OX40 Ab group, and n=9 mice in PL1-OX40+anti-OX40 Ab group. Statistical significance in c was analyzed with two-way ANOVA. *P < 0.05; ****P < 0.0001. Source data are provided as a Source Data file.

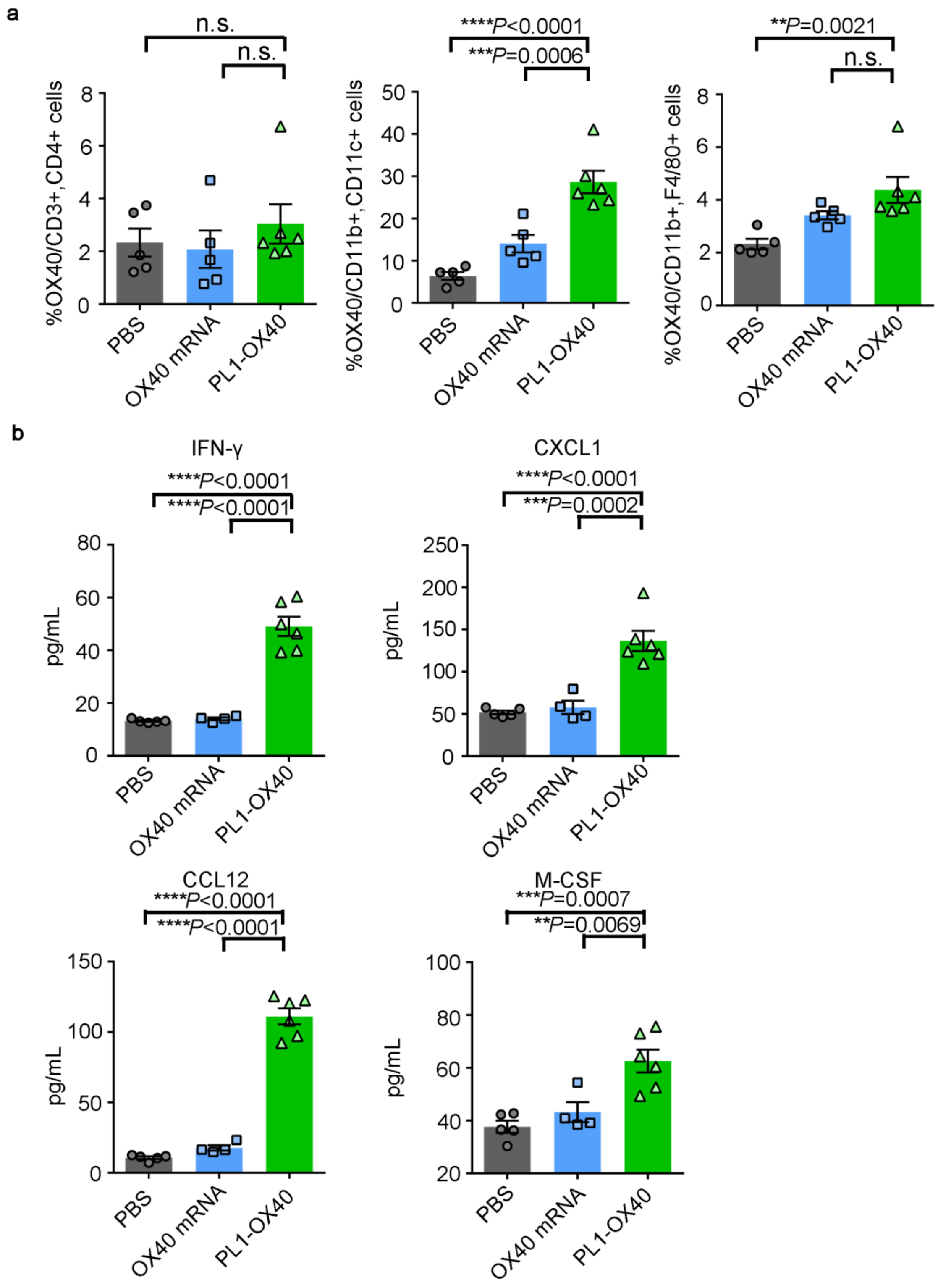

Supplementary Figure 11. Analysis of immune cell populations and cytokine levels. a, OX40 expression on the surface of CD4+ T cells, macrophages and dendritic cells after a single

intratumoral injection with PBS (n=5), OX40 mRNA (n=5) or PL1-OX40 mRNA (n=6) in A20 tumor microenvironment. **b**, Mouse plasma cytokine levels after a single intratumoral injection of PBS (n=5), OX40 mRNA (n=4) or PL1-OX40 mRNA (n=6). Data are presented as the mean ± S.E.M. Statistical significance in **a** and **b** were analyzed using one-way ANOVA followed by Dunnett's multiple comparisons test. $^{**}P < 0.01$; $^{***}P < 0.001$; $^{****}P < 0.0001$; n.s., not significant. Source data are provided as a Source Data file.

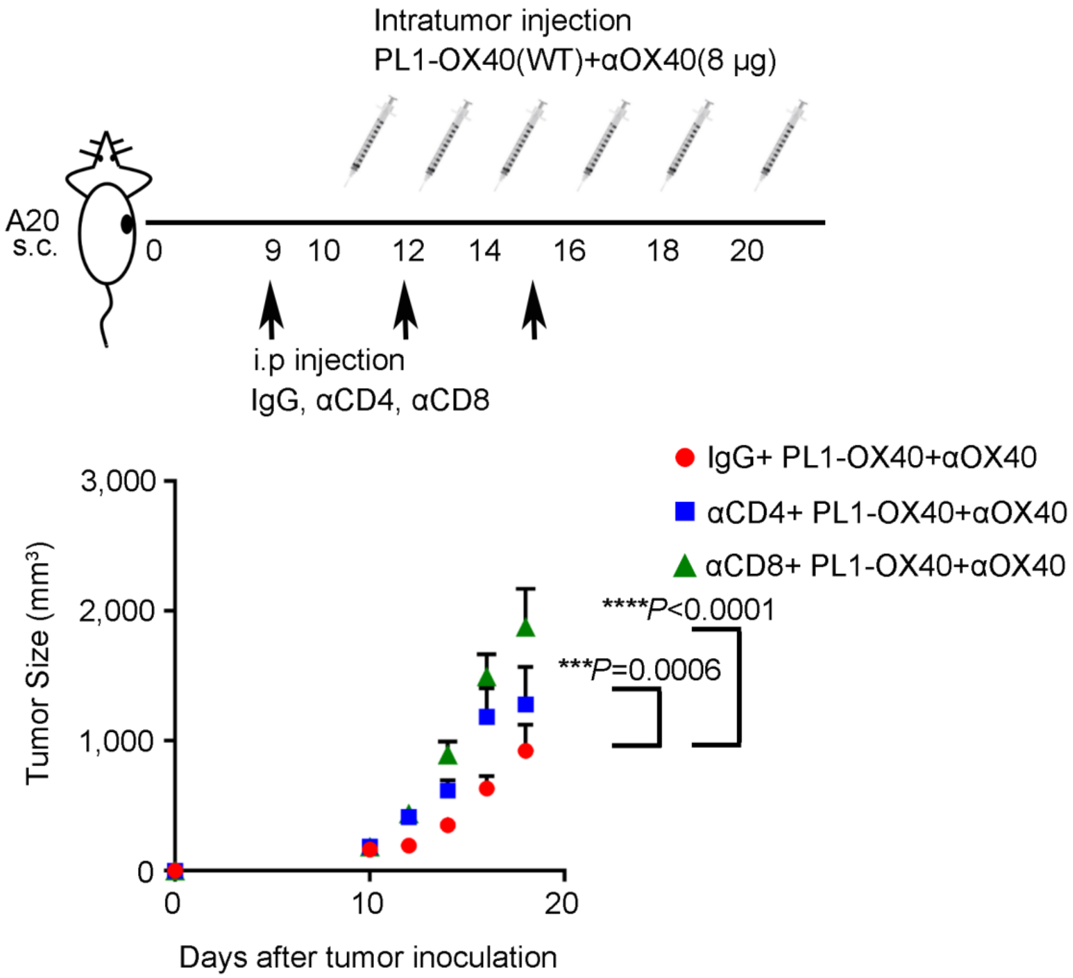

Supplementary Figure 12. Effects of CD4+ or CD8+ T cell depletion on immunotherapy of PL1-OX40+anti-OX40 Ab treatment. Schematic illustration of the A20 mouse tumor model and the treatment regimen. PL1-OX40 (10 μg mRNA/mouse), anti-OX40 Ab (8 μg/mouse), CD8α (200 μg/mouse), and CD4 (200 μg/mouse). Tumor volumes of IgG + PL1-OX40 + anti-OX40 (n=9), anti-mouse CD8α + PL1-OX40 + anti-OX40 (n=9), or anti-mouse CD4 + PL1-OX40 + anti-OX40 (n=9). Data are presented as the mean ± S.E.M. Statistical significance was analyzed with two-way ANOVA. **P < 0.01; ****P < 0.0001. Source data are provided as a Source Data file.

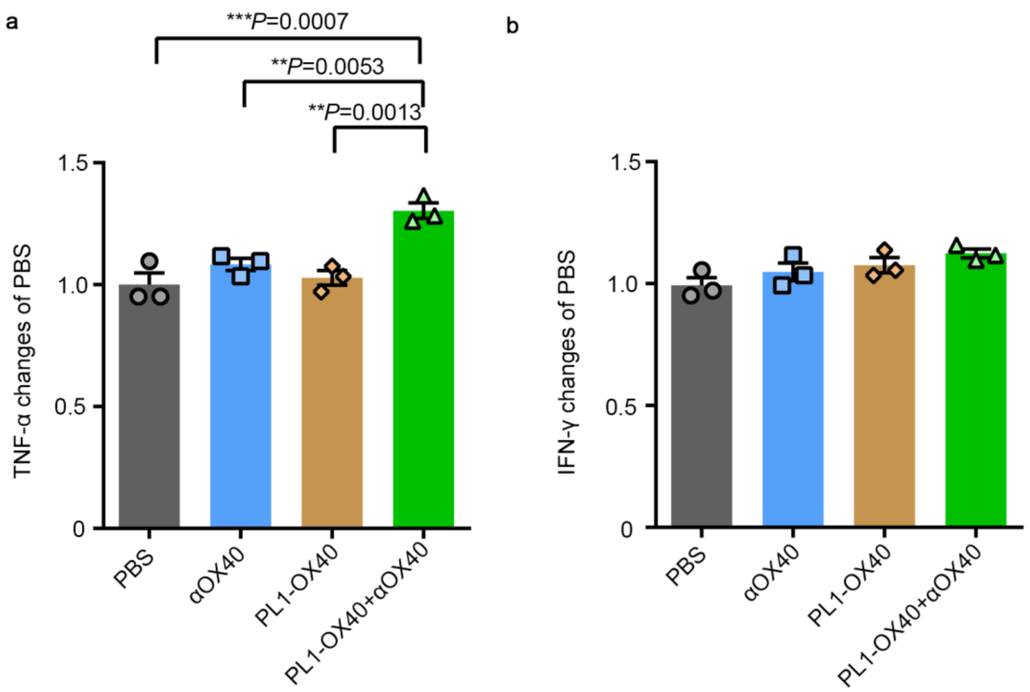

Supplementary Figure 13. Cytokines production from primary T cells *ex vivo*. Primary T cells were isolated from mouse spleen and treated with PBS, anti-OX40 Ab, PL1-OX40, or PL1-OX40 + anti-OX40 Ab. Intracellular TNF-α (**a**) and IFN-γ (**b**) levels were tested by ELISA after 24 hours. Results are representative of three independent experiments. Data are presented as the mean ± S.E.M. Statistical significance were analyzed using one-way ANOVA followed by Dunnett's multiple comparison test. $^{**}P < 0.01$; $^{***}P < 0.001$. Source data are provided as a Source Data file.

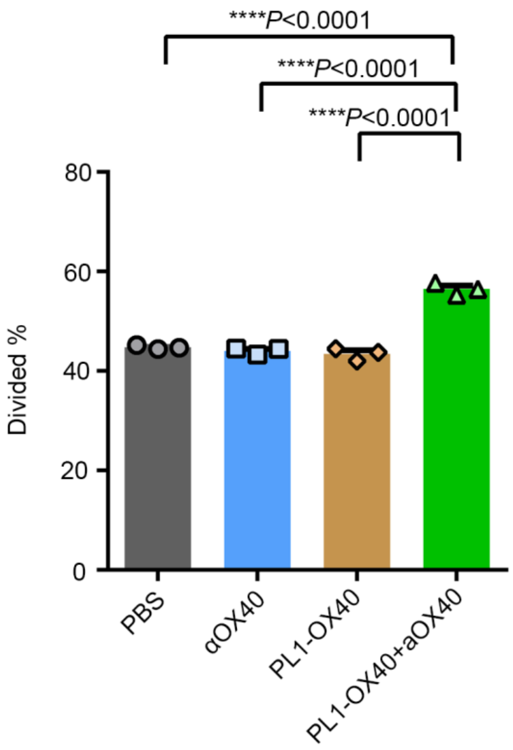

Supplementary Figure 14. Proliferation of primary T cells *ex vivo*. Primary T cells were isolated from mouse spleen and treated with PBS, anti-OX40 Ab, PL1-OX40, or PL1-OX40 + anti-OX40 Ab. 60 hours after the treatment, cell division was analyzed using carboxyfluorescein succinimidyl ester (CFSE) staining on a flow cytometer. Results are representative of three independent experiments. Data are presented as the mean ± S.E.M. Statistical significance were analyzed using one-way ANOVA followed by Dunnett's multiple comparison test. ****P < 0.0001. Source data are provided as a Source Data file.

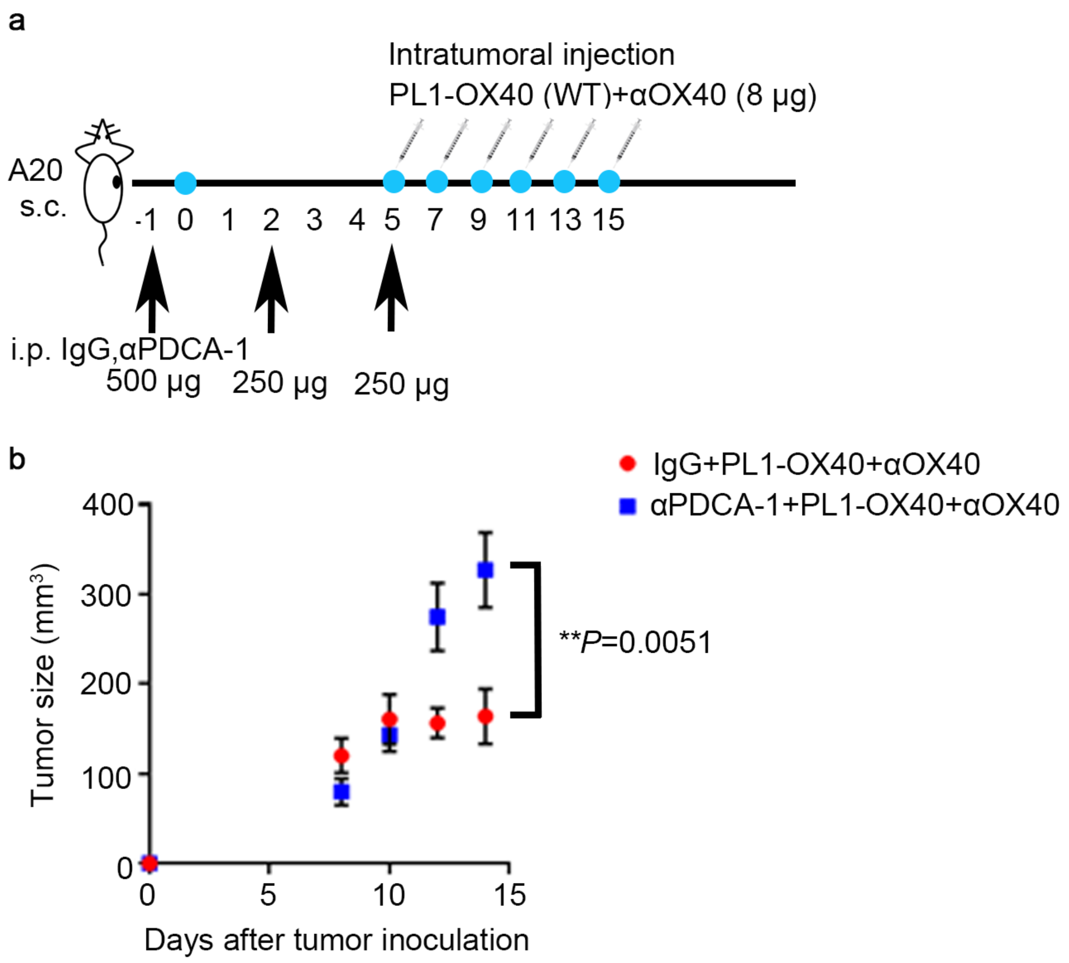

Supplementary Figure 15. Effects of DCs depletion on the treatment of PL1-OX40+anti-OX40 Ab treatment. a, Schematic illustration of the A20 mouse tumor model and the treatment regimen. **b**, Tumor volumes of IgG + PL1-OX40 + anti-OX40 (n=10) and anti-PDCA-1 + PL1-OX40 + anti-OX40 (n=10). Data are presented as the mean ± S.E.M. Statistical significance was analyzed with two-way ANOVA. ***P* < 0.01. Source data are provided as a Source Data file.

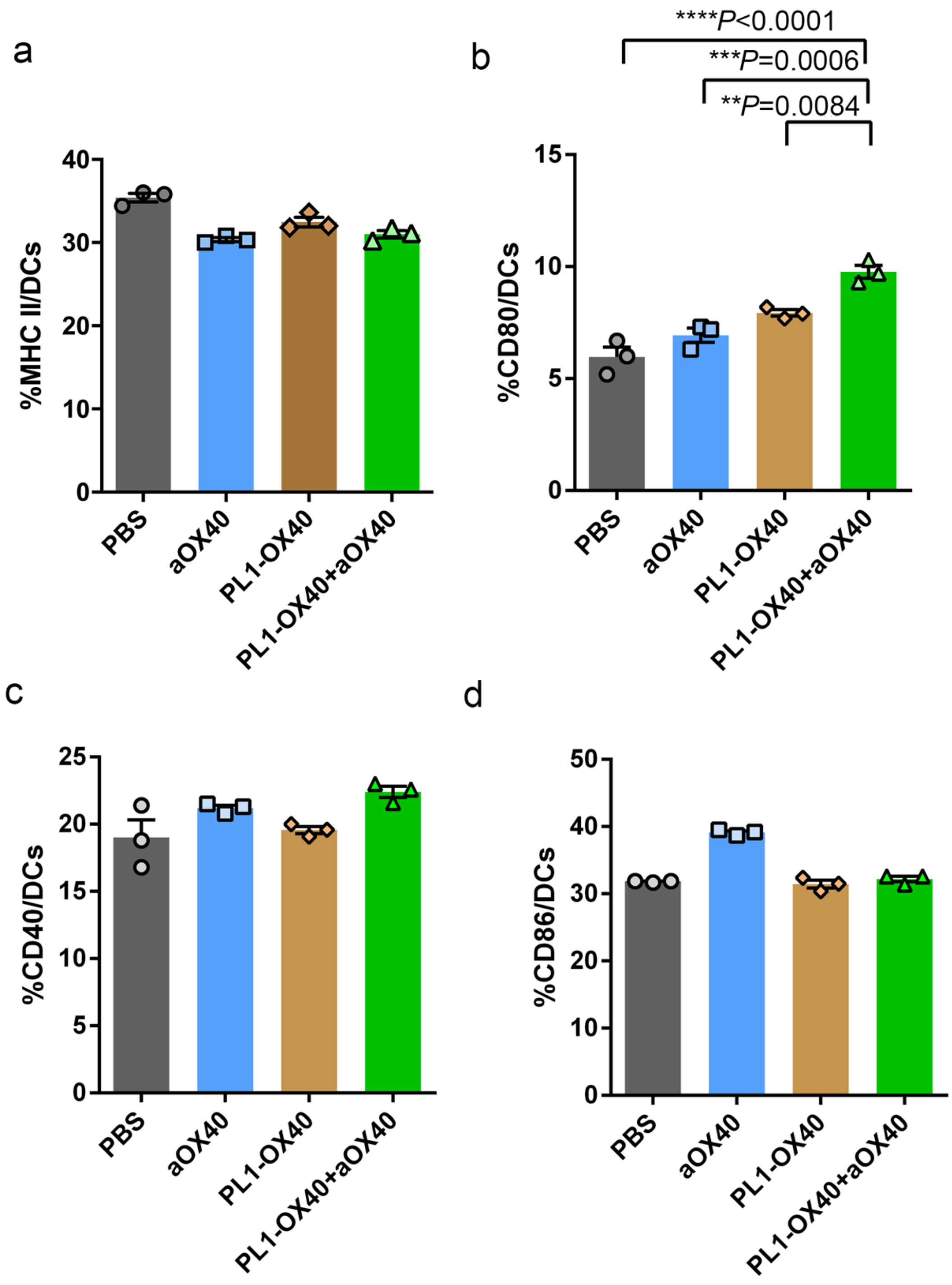

Supplementary Figure 16. Expression of surface markers on DCs *ex vivo*. Primary DCs were treated with PBS, anti-OX40 Ab, PL1-OX40, or PL1-OX40 + anti-OX40 Ab. **a-d**, Expression of MHC II (**a**), CD80 (**b**), CD40 (**c**), and CD86 (**d**). Data are presented as the mean ± S.E.M. Results are representative of three independent experiments. Statistical significance was analyzed using

one-way ANOVA followed by Dunnett's multiple comparison test. $^{**}P < 0.01$; $^{***}P < 0.001$; $^{****}P < 0.0001$. Source data are provided as a Source Data file.

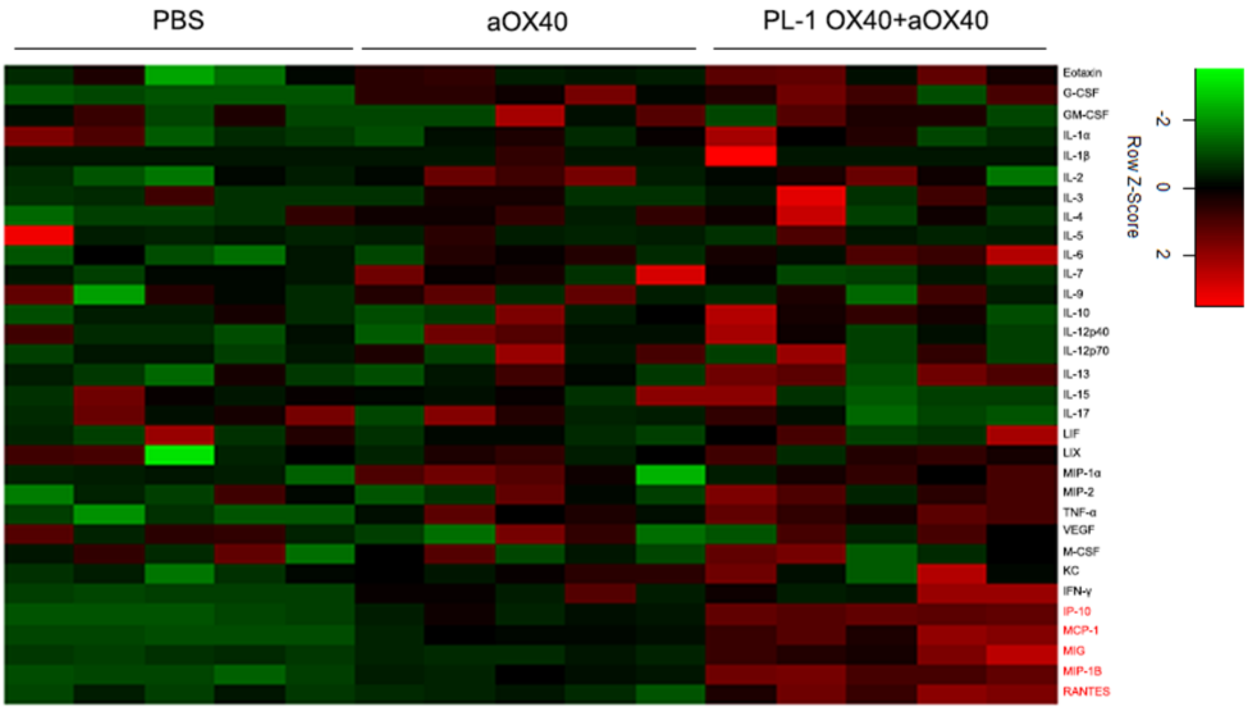

Supplementary Figure 17. Cytokines and chemokines levels in plasma from A20 tumor-bearing mouse after one-time treatment. The A20 tumor-bearing mouse were i.t. injected with PBS (n=5), anti-OX40 (n=5), or PL1-OX40+ anti-OX40 (n=5). After one day, the plasma was analyzed for mouse cytokines and chemokines. Row data are normalized by calculating the standard score (Z-score) in R3.4.3 (www.r-project,org). Z-score = (protein level in each sample-row mean)/row standard deviation (S.D.). Source data are provided as a Source Data file.

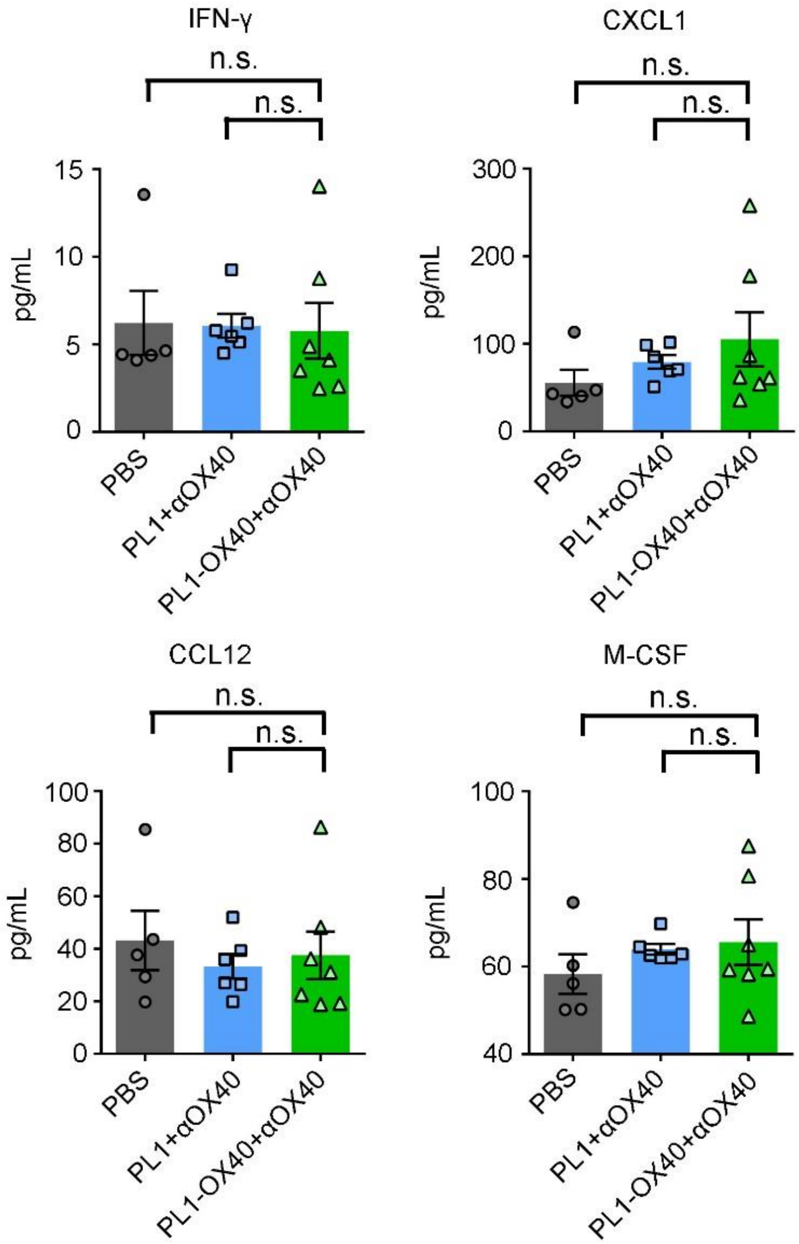

Supplementary Figure 18. The plasma cytokines after six doses of intratumoral treatment. PBS (n=5), PL1+anti-OX40 (n=6), and PL1-OX40+ anti-OX40 (n=6). Data are presented as the mean ± S.E.M. Statistical significance was analyzed using one-way ANOVA followed by Dunnett's multiple comparison test. n.s., not significant. Source data are provided as a Source Data file.

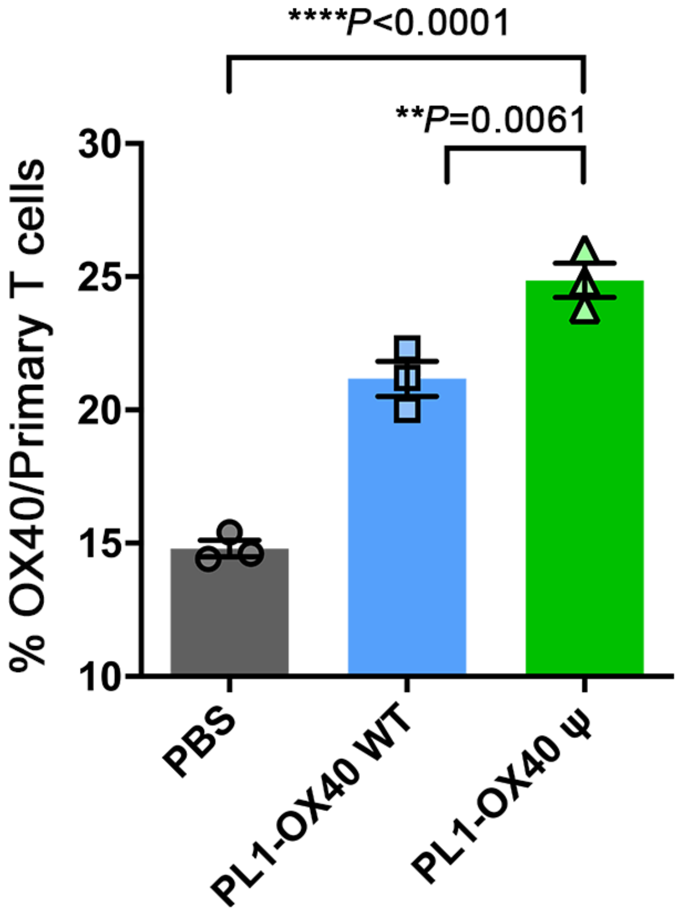

Supplementary Figure 19. PL1-OX40 (WT) and PL1-OX40 (ψ, pseudouridine-5′-triphosphate) induced OX40 expression in primary T cells. Data are presented as the mean ± S.E.M. Results are representative of three independent experiments. Statistical significance was analyzed using one-way ANOVA followed by Dunnett's multiple comparison test. $**P < 0.01$; $****P < 0.0001$. Source data are provided as a Source Data file.

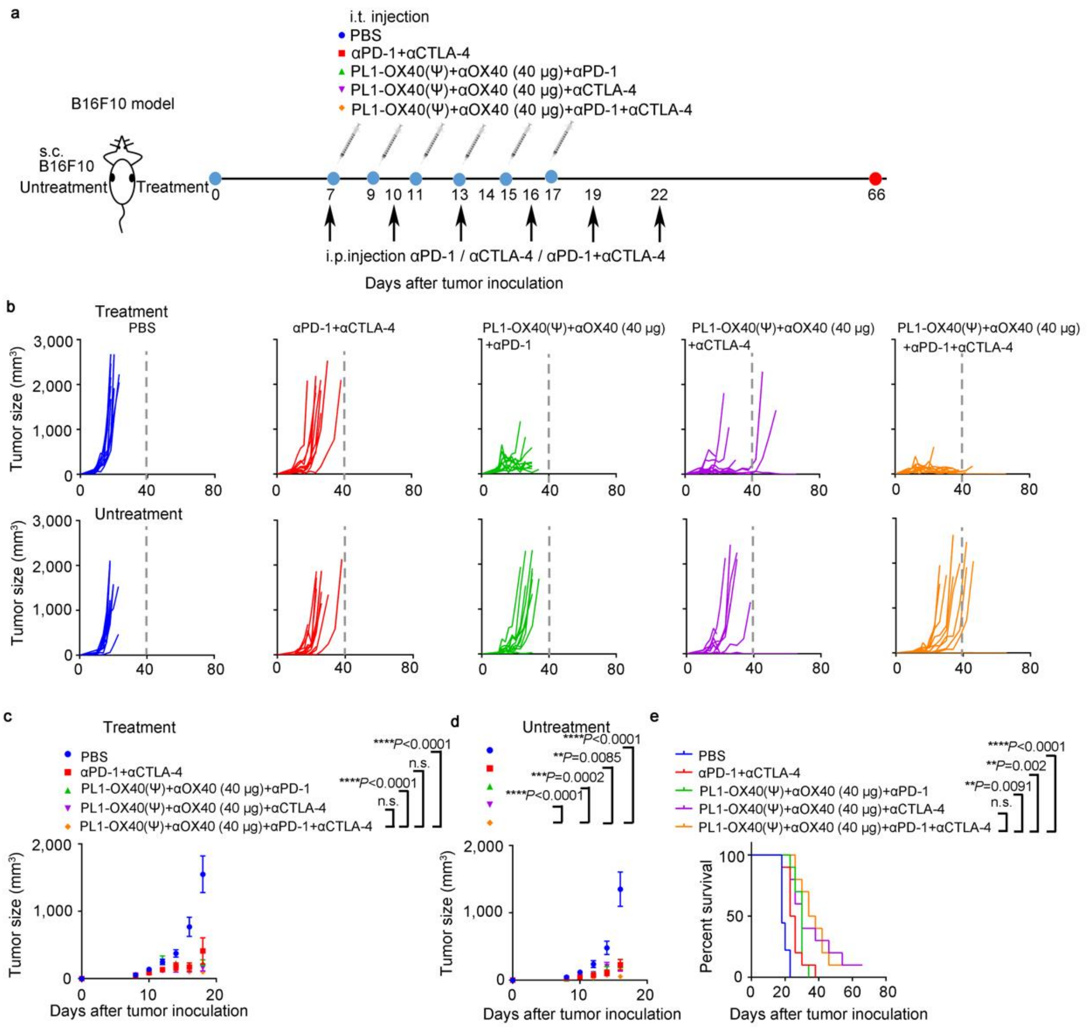

Supplementary Figure 20. Two-side tumor model of B16F10 tumor. a, Schematic illustration of the treatment. **b**, Tumor volumes of individual mice with treated and untreated tumors (n=10 per group) following six doses of PBS, anti-PD-1 + anti-CTLA-4, PL1-OX40(ψ, pseudouridine-5′-triphosphate) + anti-OX40 (40 μg) + anti-PD-1, PL1-OX40(ψ) + anti-OX40 (40 μg) + anti-CTLA-4, or PL1-OX40(ψ) + anti-OX40 (40 μg) + anti-PD-1 +anti-CTLA-4. anti-PD-1 (100 μg) and/or anti-CTLA-4 (100 μg) were i.p injected for six doses. **c**, **d**, **e** Tumor volumes of the treated side (**c**), Tumor volumes of the untreated side (**d)** and survival of mice (**e**). Data in **c**, **d**, and **e** are presented as the mean ± S.E.M. Statistical significance in **c** and **d** was analyzed with two-way ANOVA.

Statistical significance in **e** was analyzed using the log-rank (Mantel–Cox) test. $**P < 0.01$; $***P < 0.001$; $****P < 0.0001$; n.s., not significant. Source data are provided as a Source Data file.

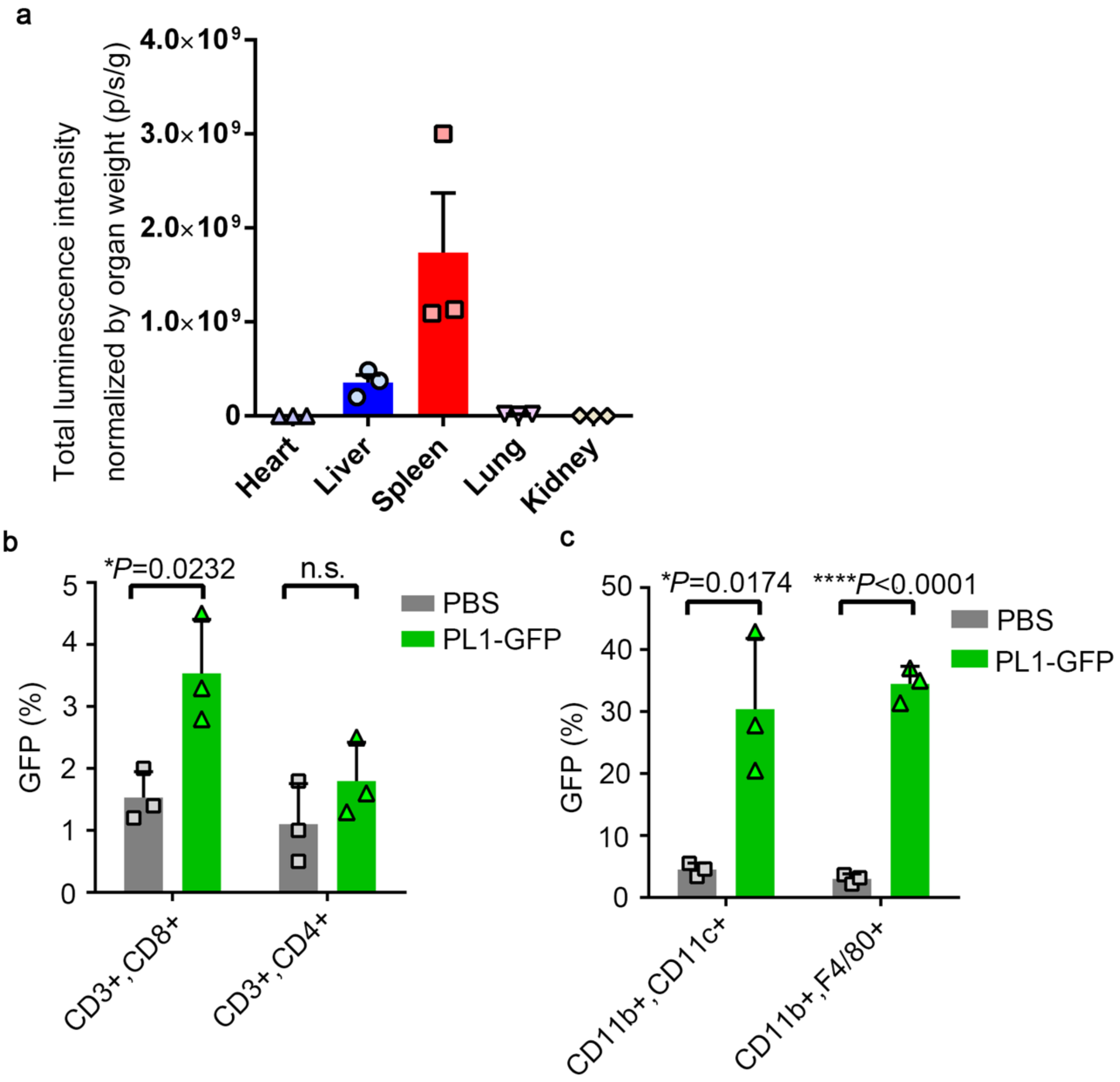

Supplementary Figure 21. a, Quantification of luminescence intensity after i.v. injection of PL1-Fluc luciferase mRNA (n = 3). **b**, **c**, GFP expression in various cell types from mouse spleen after i.v. injection of PL1-GFP in C57BL/6 mice. Data in b and c are presented as the mean ± S.E.M (n = 3). Statistical significance in b and c was analyzed using two-tailed Student's *t* test. **P* < 0.05; *****P* < 0.0001; n.s., not significant. Source data are provided as a Source Data file.

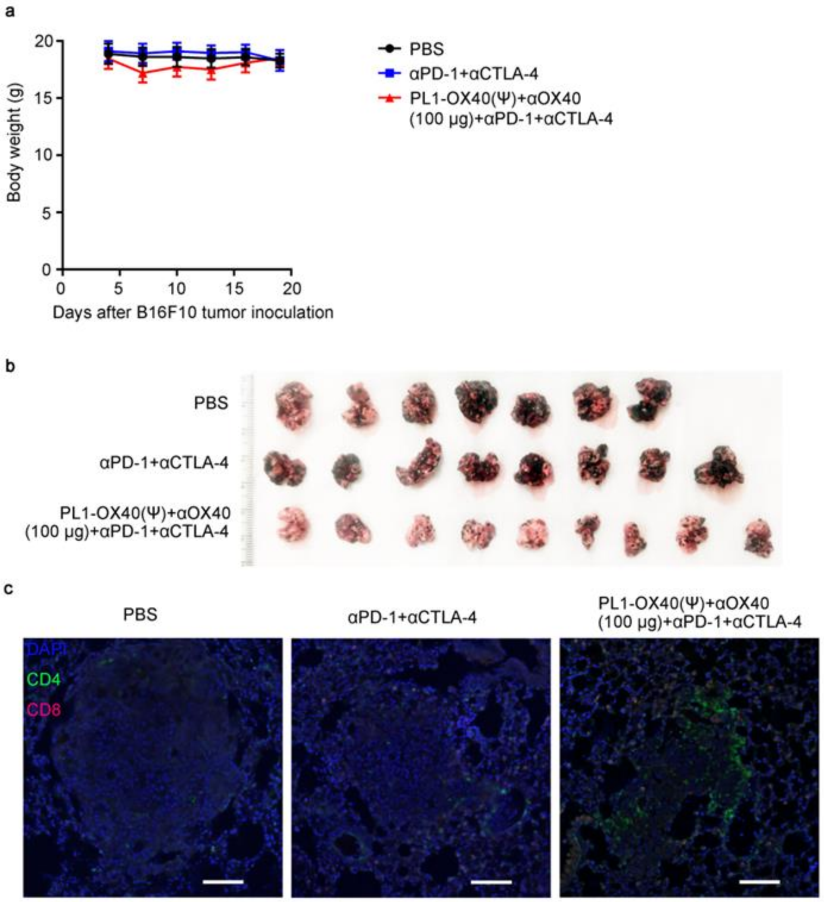

Supplementary Figure 22. Antitumor efficacy in a lung metastasis mouse model. 2×10^5 B16F10 cells were intravenously injected into C57BL/6 mice. Mice received i.p. injections of PBS (n=7), i.p. injections anti-mouse PD-1 (100 μg) + anti-mouse CTLA-4 (100 μg) Abs (n=8), or i.v. injections of PL1-OX40 (ψ, pseudouridine-5′-triphosphate) + i.p. injections of anti-OX40 (100 μg) + i.p. injections of anti-PD-1 (100 μg) + anti-CTLA-4 (100 μg) (n=9) every three days. **a**, Mouse body weight. Data are present as the mean ± SD. **b**, Images of melanoma metastasis in the mouse lungs at day 19 after i.v. injection of B16F10 cells. **c**, Representative CD4+ and CD8+ T cell staining in mouse lung tumor sections in the groups of PBS, anti-PD-1+ anti-CTLA-4 Abs, and PL1-OX40 (ψ) + anti-OX40 (100 μg) + anti-PD-1 + anti-CTLA-4 Abs. Scale bars = 100 μm. Data were shown from one representative mouse (n=1) and three independent fields of view . Source data are provided as a Source Data file.

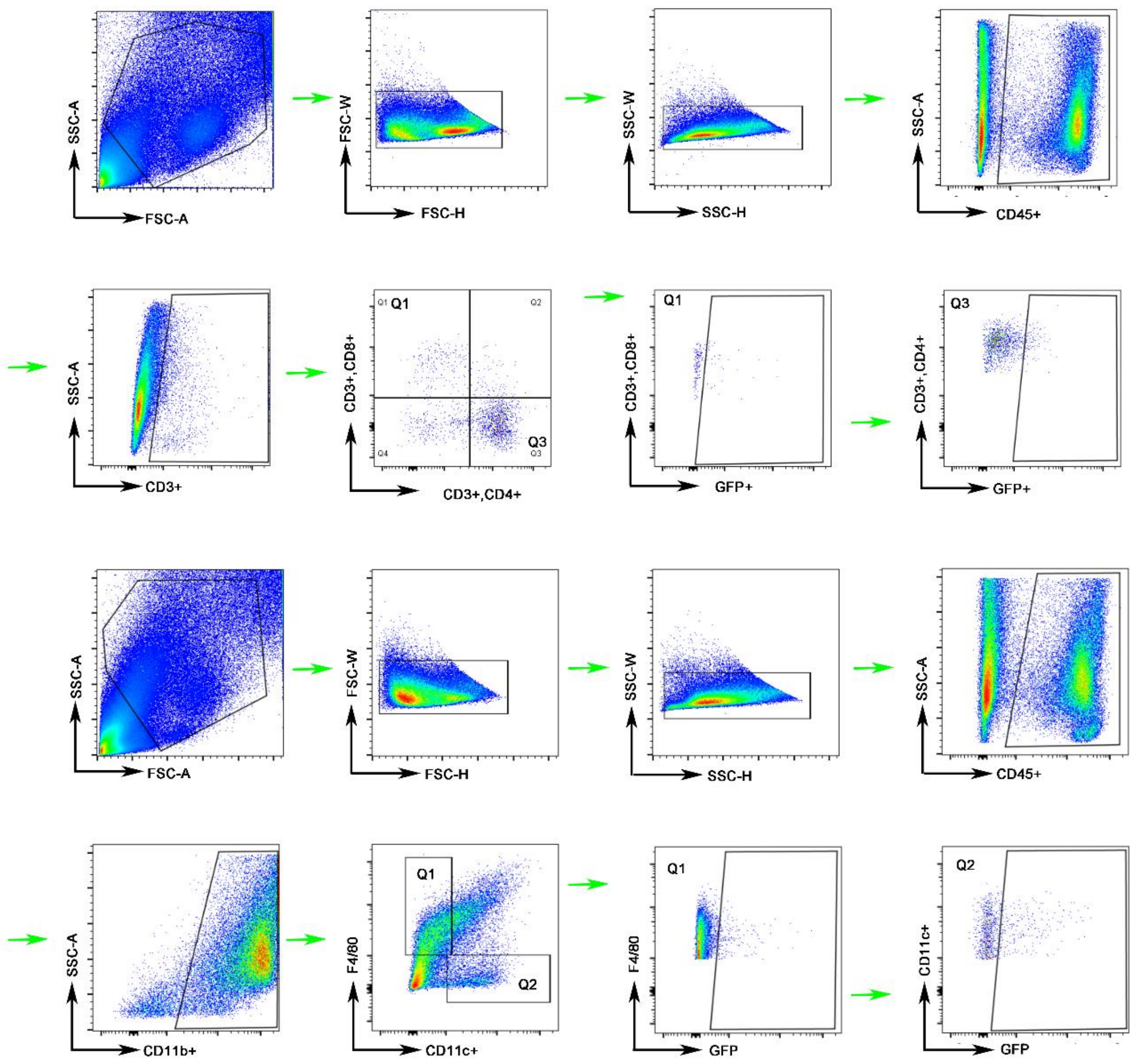

Supplementary Figure 23. Flow Cytometry Gating strategy. This strategy was applied in Fig 2g, 3f, 3h, Fig 5d-f, Supplementary Figure 7d-g, and Supplementary Figure 11a. Cells were first gated on FSC/SSC to define single cells. Then, gate CD45 positive cells, CD3 positive cells, CD4/CD8 positive cells and OX40/GFP positive cells. Also, gate CD45 positive cells, CD11b positive cells, CD11c/F4/80 positive cells and OX40/GFP positive cells.

Supplementary References

1 Anderson, M. A., Shim, H., Raushel, F. M. & Cleland, W. W. Hydrolysis of phosphotriesters: determination of transition states in parallel reactions by heavy-atom isotope effects. *J. Am. Chem. Soc*. **123**, 9246-9253 (2001).

2 Balaji, B. S. & Lewis, M. R. Double exponential growth of aliphatic polyamide dendrimers via AB(2) hypermonomer strategy. *Chem. Commun. (Camb)* **30**, 4593-4595, (2009).
