# Supplementary material for: Biomimetic nanoparticles deliver mRNAs encoding costimulatory receptors and enhance T cell mediated cancer immunotherapy
Source: Nat Commun. 2021 Dec 14;12:7264. doi: 10.1038/s41467-021-27434-x (PMC8671507; doi:10.1038/s41467-021-27434-x)
Supplement: Supplementary file 2 — Reporting Summary [file 41467_2021_27434_MOESM2_ESM.pdf]

nature portfolio

Corresponding author(s): Yizhou Dong
Last updated by author(s): Nov 12, 2021

Reporting Summary

Nature Portfolio wishes to improve the reproducibility of the work that we publish. This form provides structure for consistency and transparency in reporting. For further information on Nature Portfolio policies, see our Editorial Policies and the Editorial Policy Checklist.

Statistics

For all statistical analyses, confirm that the following items are present in the figure legend, table legend, main text, or Methods section.

n/a	Confirmed	
☐	☒	The exact sample size (*n*) for each experimental group/condition, given as a discrete number and unit of measurement
☐	☒	A statement on whether measurements were taken from distinct samples or whether the same sample was measured repeatedly
☐	☒	The statistical test(s) used AND whether they are one- or two-sided<br>*Only common tests should be described solely by name; describe more complex techniques in the Methods section.*
☐	☒	A description of all covariates tested
☐	☒	A description of any assumptions or corrections, such as tests of normality and adjustment for multiple comparisons
☐	☒	A full description of the statistical parameters including central tendency (e.g. means) or other basic estimates (e.g. regression coefficient) AND variation (e.g. standard deviation) or associated estimates of uncertainty (e.g. confidence intervals)
☐	☒	For null hypothesis testing, the test statistic (e.g. *F*, *t*, *r*) with confidence intervals, effect sizes, degrees of freedom and *P* value noted<br>*Give P values as exact values whenever suitable.*
☒	☐	For Bayesian analysis, information on the choice of priors and Markov chain Monte Carlo settings
☒	☐	For hierarchical and complex designs, identification of the appropriate level for tests and full reporting of outcomes
☒	☐	Estimates of effect sizes (e.g. Cohen's *d*, Pearson's *r*), indicating how they were calculated

Our web collection on statistics for biologists contains articles on many of the points above.

Software and code

Policy information about availability of computer code

Data collection	LSRFortessa Flow Cytometer (BD Biosciences), LSR II Flow Cytometer (BD Biosciences), SpectraMax M5 microplate reader. Microsoft Excel (Version 2106).
Data analysis	GraphPad Prism 7, R-project (R3.4.3), Microsoft Excel (Version 2106), and FlowJo (Version 10.4).

For manuscripts utilizing custom algorithms or software that are central to the research but not yet described in published literature, software must be made available to editors and reviewers. We strongly encourage code deposition in a community repository (e.g. GitHub). See the Nature Portfolio guidelines for submitting code & software for further information.

Data

Policy information about availability of data

All manuscripts must include a data availability statement. This statement should provide the following information, where applicable:

- Accession codes, unique identifiers, or web links for publicly available datasets
- A description of any restrictions on data availability
- For clinical datasets or third party data, please ensure that the statement adheres to our policy

The datasets generated during and/or analyzed during the current study are available from the corresponding author on reasonable request.

Field-specific reporting

Please select the one below that is the best fit for your research. If you are not sure, read the appropriate sections before making your selection.

☒ Life sciences ☐ Behavioural & social sciences ☐ Ecological, evolutionary & environmental sciences

For a reference copy of the document with all sections, see nature.com/documents/nr-reporting-summary-flat.pdf

Life sciences study design

All studies must disclose on these points even when the disclosure is negative.

Sample size	Standard number of replication is 3 in all in vitro studies. A minimum of 3 biological repeats is applied to evaluate significance. A minimum of 4 mice were used for in vivo studies and details regarding sample size of in vivo studies are provided in the methods section and figure legends. The sample size was selected to minimize the number of animals and meanwhile obtain statistical validity based on power analysis.
Data exclusions	No data were excluded.
Replication	Most experiments were repeated 2-3 times, only DC depletion and double-sided tumor experiments were performed once.
Randomization	Samples were randomly allocated to corresponding experimental groups.
Blinding	The synthesis of phospholipid and glycolipid derivatives, mRNA preparation, FACS, and animal experiments were performed by multiple researchers, who had minimal information of sample identification. However, samples were not formally blinded.

Reporting for specific materials, systems and methods

We require information from authors about some types of materials, experimental systems and methods used in many studies. Here, indicate whether each material, system or method listed is relevant to your study. If you are not sure if a list item applies to your research, read the appropriate section before selecting a response.

Materials & experimental systems

n/a	Involved in the study
☐	☒ Antibodies
☐	☒ Eukaryotic cell lines
☒	☐ Palaeontology and archaeology
☐	☒ Animals and other organisms
☒	☐ Human research participants
☒	☐ Clinical data
☒	☐ Dual use research of concern

Methods

n/a	Involved in the study
☒	☐ ChIP-seq
☐	☒ Flow cytometry
☒	☐ MRI-based neuroimaging

Antibodies

Antibodies used	Antibodies: anti-OX40 antibody (InVivoPlus anti-mouse OX40 (CD134), BioXcell, Cat: BP0031), anti-CD137 antibody (InVivoPlus antimouse 4-1BB (CD137), BioXcell, Cat: BP0169). Antibodies were diluted to the desired concentration before use.<br>Mouse fluorescently conjugated antibodies at a 1:200 dilution used for FACS: anti-CD45 APC (30-F11) (Thermo Fisher, Cat: # 17-0451-82; dilution), anti-CD3 PE (145-2C11) (BD Pharmingen, Cat: # 553063), anti-CD8 APC-efluor 780 (53-4.7) (Thermo Fisher, Cat: # 47-0081-82), anti-F4/80 PE-eFluor 610 (BM8) (Thermo Fisher, Cat: # 61-4801-82), anti-CD11c PE-Cyanine7 (N418) (Thermo Fisher, Cat: # 25-0114-82), anti-Foxp3-PEeFluor ® 610 (FJK-16s) (Thermo Fisher, Catalog # 61-5773-82).<br>Mouse fluorescently conjugated antibodies at a 1:100 dilution used for FACS: anti-CD137-FITC (1AH2) (BD Pharmingen, Catalog No. 558975) and anti-OX40 FITC (OX86) (Abcam, Cat: # ab33998).<br>Mouse fluorescently conjugated antibodies at a 1:50 dilution used for FACS: anti-CD4 Pacific Blue (RM4-5) (Thermo Fisher Cat: # MCD0428), anti-CD11b Pacific Blue (M1/70.15) (Thermo Fisher, Cat: # RM2828).
Validation	Anti-OX40 and anti-CD137 monoclonal antibodies were verified by the supplier.<br>Anti-CD45,CD3,CD4,CD8,CD11b,CD11c,F4/80, anti-Foxp3, anti-CD137-FITC, anti-OX40-FITC monoclonal antibodies were validated by the supplier under tested dilutions in mouse and have been reported for use in flow cytometric analysis.<br>Filtration: 0.2 µm post-manufacturing filtered.

Eukaryotic cell lines

Policy information about cell lines

Cell line source(s)	E.G7-OVA, A20, CT26 cell lines were obtained from ATCC. B16F10 cell line was obtained from the lab of Dr. Jianhua Yu.

Authentication	Cell lines were not independently authenticated.
Mycoplasma contamination	Cell lines were not independently tested for mycoplasma contamination.
Commonly misidentified lines (See ICLAC register)	No commonly misidentified cell lines were used.

Animals and other organisms

Policy information about studies involving animals; ARRIVE guidelines recommended for reporting animal research

Laboratory animals	Female C57BL/6 , BALB/c mice (6–12 weeks) were purchased from Jackson Lab. All animals were housed in single-unit cages with 12-hour alternate light and dark cycles and at controlled ambient temperature (68-79 F) with humidity between 30%-70%.
Wild animals	The study did not involve wild animals.
Field-collected samples	The study did not involve samples collected from field.
Ethics oversight	All mouse experiments were carried out in accordance with the protocols approved by the Institutional Animal Care and Use Committee (IACUC) of the Ohio State University.

Note that full information on the approval of the study protocol must also be provided in the manuscript.

Flow Cytometry

Plots

Confirm that:

☒ The axis labels state the marker and fluorochrome used (e.g. CD4-FITC).

☒ The axis scales are clearly visible. Include numbers along axes only for bottom left plot of group (a 'group' is an analysis of identical markers).

☒ All plots are contour plots with outliers or pseudocolor plots.

☒ A numerical value for number of cells or percentage (with statistics) is provided.

Methodology

Sample preparation	TIL cells analysis: the tumors mechanically dissociated with gentle MACS dissociator, and digested in Tumor Dissociation Kit, Mouse (MACS, 130-096-730) according to the protocol. Cells were diluted in PBS containing 2% FBS for staining with fluorescent antibodies. The TIL cell populations were analysis on a BD LSR Fortessa or BD LSR II flow cytometer (BD Biosciences).
Instrument	flow cytometer (LSRII, BD; Fortessa, BD)
Software	FlowJo (Version 10.4)
Cell population abundance	B16F10 control tumors: CD45+/CD3+ in CD45+ (~6.3%), CD3+/CD8+ in CD45+/CD3+ (~11.3%), CD3+/CD4+ in CD45+/CD3+(~49.7%) ;CD45+/CD11b+ in CD45+(~64.9%), CD11b+/CD11c+ in CD45+/CD11b+(~8.2%), CD11b+/F4/80+ in CD45+/CD11b+(16.9~%).
Gating strategy	Cells were first gated on FSC/SSC to define single cells. Then, gate CD45 positive cells, CD3 positive cells, CD4/CD8 positive cells and OX40/GFP positive cells. Also, gate CD45 positive cells, CD11b positive cells, CD11c/F4/80 positive cells and OX40/GFP positive cells.

☒ Tick this box to confirm that a figure exemplifying the gating strategy is provided in the Supplementary Information.
